# Metagenomics-assembled genomes reveal microbial metabolic adaptation to athalassohaline environment, the case Lake Barkol, China

**DOI:** 10.3389/fmicb.2025.1550346

**Published:** 2025-06-04

**Authors:** Maripat Xamxidin, Xuanqi Zhang, Gang Zheng, Can Chen, Min Wu

**Affiliations:** ^1^College of Life Sciences, Zhejiang University, Hangzhou, China; ^2^College of Architecture and Engineering, Zhejiang University, Hangzhou, China; ^3^Ocean Research Center of Zhoushan, Zhejiang University, Zhoushan, China

**Keywords:** metagenome-assembled genomes, microbial consortia, metabolic adaptation, athalassohaline Lake Barkol, osmoadaptation strategies

## Abstract

Salt-tolerant and halophilic microorganisms are critical drivers of ecosystem stability and biogeochemical cycling in athalassohaline environments. Lake Barkol, a high-altitude inland saline lake, provides a valuable natural setting for investigating microbial community dynamics and adaptation mechanisms under extreme salinity. In this study, we employed high-throughput metagenomic sequencing to characterize the taxonomic composition, metabolic potential, and ecological functions of microbial communities in both water and sediment samples from Lake Barkol. We reconstructed 309 metagenome-assembled genomes (MAGs), comprising 279 bacterial and 30 archaeal genomes. Notably, approximately 97% of the MAGs could not be classified at the species level, indicating substantial taxonomic novelty in this ecosystem. Dominant bacterial phyla included *Pseudomonadota*, *Bacteroidota*, *Desulfobacterota*, *Planctomycetota*, and *Verrucomicrobiota*, while archaeal communities were primarily composed of *Halobacteriota*, *Thermoplasmatota*, and *Nanoarchaeota*. Metabolic reconstruction revealed the presence of diverse carbon fixation pathways, including the Calvin-Benson-Bassham (CBB) cycle, the Arnon-Buchanan reductive tricarboxylic acid (rTCA) cycle, and the Wood-Ljungdahl pathway. Autotrophic sulfur-oxidizing bacteria, alongside members of *Cyanobacteria* and *Desulfobacterota*, were implicated in primary production and carbon assimilation. Nitrogen metabolism was predominantly mediated by *Gammaproteobacteria*, with evidence for both nitrogen fixation and denitrification processes. Sulfur cycling was largely driven by *Desulfobacterota* and *Pseudomonadota*, contributing to sulfate reduction and sulfur oxidation pathways. Microbial communities exhibited distinct osmoadaptation strategies. The “salt-in” strategy was characterized by ion transport systems such as Trk/Ktr potassium uptake and Na^+^/H^+^ antiporters, enabling active intracellular ion homeostasis. In contrast, the “salt-out” strategy involved the biosynthesis and uptake of compatible solutes including ectoine, trehalose, and glycine betaine. These strategies were differentially enriched between water and sediment habitats, suggesting spatially distinct adaptive responses to local salinity gradients and nutrient regimes. Additionally, genes encoding microbial rhodopsins were widely distributed, suggesting that rhodopsin-based phototrophy may contribute to supplemental energy acquisition under osmotic stress conditions. The integration of functional and taxonomic data highlights the metabolic versatility and ecological roles of microbial taxa in sustaining biogeochemical processes under hypersaline conditions. Overall, this study reveals extensive taxonomic novelty and functional plasticity among microbial communities in Lake Barkol and underscores the influence of salinity in structuring microbial assemblages and metabolic pathways in athalassohaline ecosystems.

## 1 Introduction

Hypersaline environments, including solar salterns and athalassohaline lakes, are characterized by salinity levels exceeding that of seawater (~35 g/L) and represent important ecosystems for understanding microbial adaptation to extreme osmotic stress, particularly under conditions ranging from 100 g/L to halite saturation (Saccò et al., 2021). The ecological dynamics in these extreme habitats are predominantly governed by halophilic microbial communities that have evolved specialized mechanisms to survive under such harsh conditions. Metagenomics, a culture-independent approach first introduced by Handelsman et al. (1998), enables the investigation of microbial communities *in situ* without the need for cultivation, allowing the exploration of their functional potential, including genes and metabolic pathways critical for environmental adaptation (Riesenfeld et al., 2004). Extensive metagenomic studies have revealed surprising microbial diversity and niche-specific adaptations across diverse hypersaline habitats (Liu et al., 2023), and this approach has proven particularly transformative in studying extremophiles inhabiting hypersaline ecosystems (Braga et al., 2016; La Cono et al., 2024).

For instance, metagenomic analyses of Lake Hillier in Australia, known for its characteristic pink coloration, uncovered a community of polyextremophiles that produces bacterioruberin and other carotenoids as a response to combined salinity and UV stress (Sierra et al., 2022). Similarly, in the acidic hypersaline lakes of the Yilgarn Craton, microbial communities were found to perform complex sulfur oxidation pathways, offering insights into early Martian biogeochemistry (Johnson et al., 2015). Studies in hypersaline soda lakes of the Kulunda Steppe and Xinjiang have highlighted the role of ionic composition in shaping microbial function, revealing haloalkaliphiles that synthesize glycine betaine for osmoprotection (Vavourakis et al., 2016), as well as salinity-dependent dynamics of antimicrobial resistance genes associated with *Halomonas* and *Salinibacter* (Liang et al., 2021). Notably, metagenomic sequencing of Siberian soda lake sediments provided the first genomic evidence for Candidate Phyla Radiation (CPR) microbes adapted to haloalkaline conditions, expanding current understanding of the metabolic versatility of uncultured prokaryotes in sulfur disproportionation and CO_2_ fixation (Vavourakis et al., 2018).

Lake ecosystems play a central role in biogeochemical cycling, with microbial communities driving key processes that regulate nutrient dynamics (Bertrand et al., 2015). A mechanistic understanding of microbial core traits is essential for linking community composition with elemental cycling and ecosystem function (Litchman et al., 2015; Zuñiga et al., 2017). Athalassohaline lakes, distinct from thalassohaline systems derived from marine sources, typically occur in arid or semi-arid regions and are characterized by low precipitation and high evaporation rates (Mackenzie et al., 1995). In Xinjiang, China, numerous enclosed hypersaline lakes exist in such climates, where limited precipitation and intense evaporation facilitate salt accumulation, giving rise to athalassohaline conditions shaped by regional hydroclimate (Ma et al., 2004). The water balance in these lakes is sustained by groundwater recharge and seasonal river inflow, yet evaporation consistently exceeds input, resulting in high concentrations of dissolved salts and minerals (Zheng, 1987). As a result, athalassohaline lakes serve as unique natural laboratories for studying microbial diversity, adaptation strategies under extreme conditions, and the discovery of novel microbial taxa and potential biotechnological applications (Ponomarova and Patil, 2015).

Lake Barkol (43.61°N, 92.82°E), located in the arid temperate continental zone of the eastern Tianshan Mountains in Xinjiang, is a representative athalassohaline hypersaline lake. It exhibits extreme salinity levels, reaching up to 244 g/L, and is classified as a sodium sulfate-type system dominated by SO₄^2−^ and Na^+^ ions. Sulfate concentrations in lake water can reach 90.6 g/L, far exceeding those of Cl^−^ and CO₃^2−^. Sediments also contain extremely high sulfate levels, up to 303.59 mg/g (Lu et al., 2012; Liu et al., 2023, 2024; Cai et al., 2023). The hydrological regime of the lake is marked by low annual precipitation (~210 mm) and a high evaporation rate (~2,250 mm/year), contributing to its persistent hypersalinity and shallow water depth, which averages approximately ~0.6 m. Despite previous studies characterizing microbial community structure and isolating microbial strains from Lake Barkol using approaches such as geochemical profiling, 16S rRNA gene sequencing, amplicon sequencing, and metagenomic binning (Han et al., 2018; Gao et al., 2022, 2024), genome-resolved insights into adaptive traits, particularly across the water–sediment interface, remain lacking. Prior research has provided valuable insights into microbial community composition, functional potential, and key metabolic pathways involved in nitrogen and sulfur cycling across different habitats within Lake Barkol. Additionally, analyses of biosynthetic gene clusters (BGCs) have highlighted biotechnologically relevant potential.

Although prior studies have characterized microbial composition and function at Lake Barkol, genome-resolved insights into adaptive traits, particularly across the water–sediment interface, remain lacking. Given these unique physicochemical conditions, Lake Barkol provides an ideal model system for exploring genomic-level microbial adaptation strategies to extreme salinity. In this study, we employed genome-resolved metagenomics to reconstruct a comprehensive set of MAGs from both surface water and sediment samples from Lake Barkol. Our study offers foundational genomic insights into microbial adaptations to extreme hypersalinity at Lake Barkol’s water–sediment interface, identifying previously unexplored genetic pathways involved in carbon, nitrogen, and sulfur cycling as well as mechanisms underlying adaptation to high-salinity stress. These results provide a valuable genomic framework for understanding functional microbial ecology in hypersaline inland lakes and set the stage for in-depth comparative and biogeochemical analyses.

## 2 Materials and methods

### 2.1 Sample collection

Water and sediment samples were systematically collected from Lake Barkol, Xinjiang, China (Supplementary Figure S1). Three water samples were collected from distinct locations across the lake in August 2019, and three sediment samples were collected from the seasonally exposed shoreline in November 2020. Water samples (~2 L per site) were collected into sterile bottles and immediately pre-filtered through 10-μm polycarbonate (PC) membranes (Millipore, United States), followed by sequential filtration through 3-μm and 0.22-μm PC membranes. DNA for metagenomic analyses was extracted from biomass retained on the 3-μm and 0.22-μm filters. Sediment samples (0–5 cm depth) were collected after gently removing superficial debris to minimize potential surface contamination. All samples were immediately frozen on dry ice and stored at −80°C until DNA extraction.

At each sampling site, *in situ* environmental parameters, including water temperature, pH, electrical conductivity (EC), and salinity, were measured using a YSI ProDSS Multiparameter Digital Water Measurement System (YSI Inc., United States). Total organic carbon (TOC) in water samples was analyzed using a TOC analyzer via high-temperature catalytic oxidation (Shimadzu TOC-L, Japan). Total nitrogen (TN) concentrations in water samples were determined using ultraviolet spectrophotometry following Li et al. (2020). For sediment samples, TOC was determined using the combustion oxidation non-dispersive infrared absorption method (HT 501-2009, 2012) after extraction at a 1:5 soil-to-water ratio (w/v), and TN content was similarly measured via ultraviolet spectrophotometry as described by Li et al. (2020).

### 2.2 DNA extraction and metagenome sequencing

Total metagenomic DNA was extracted from water and sediment samples using the ALFA-SEQ Advanced Water DNA Kit and ALFA-Soil DNA Extraction Kit (Guangdong Magigene Biotechnology Co., Ltd., Guangzhou, China), respectively, following the manufacturer’s protocols. DNA concentration and purity were assessed using a NanoDrop 2000 spectrophotometer (Thermo Scientific, United States), with OD₂₆₀/OD₂₈₀ ratios ranging from 1.8 to 2.0 and DNA concentrations >1 μg. Metagenomic sequencing was performed on the Illumina HiSeq 2500 platform (Illumina Inc., San Diego, CA, United States) to generate 2 × 150 bp paired-end reads. Approximately 60 Gb and 30 Gb of raw data were obtained per sample for water and sediment, respectively. Adapter sequences and low-quality bases in raw reads were removed using Trimmomatic v0.36 (Bolger et al., 2014). The quality and quantity of cleaned reads were evaluated using SeqKit v0.15.0 (Liu et al., 2021) and FastQC v0.11.9 (Andrews, 2010).^1^

### 2.3 Metagenomic assembly

High-quality reads from each sample were assembled into contigs using MEGAHIT v1.1.2 (Li et al., 2015), which is optimized for complex metagenomic datasets. To ensure assembly quality, contigs shorter than 500 bp were discarded prior to downstream analyses. Gene prediction was performed on the resulting contigs using Prodigal v2.6.3 (Hyatt et al., 2010) in metagenomic mode (−p meta) to identify open reading frames (ORFs). Only complete ORFs, defined as those containing both start and stop codons, were retained. Non-redundant gene catalogs were generated by clustering predicted ORFs at 95% sequence identity using CD-HIT v4.8.1 (Li and Godzik, 2006) with parameters -c 0.95 -aS 0.9 -G 0 -g 1 -d 0. Clean reads from each sample were mapped back to the assembled contigs using BWA-MEM v0.7.17 (Li and Durbin, 2009). Resulting SAM-format mapping files were converted and sorted using SAMtools v1.10 (Li et al., 2009) to calculate contig coverage. Gene abundances were normalized and expressed as counts per million (CPM) by dividing the number of mapped reads per gene by its length, followed by normalization against the total number of mapped reads per sample, as previously described by Nayfach and Pollard (2016). Functional annotation of the non-redundant gene catalog was performed using KofamScan v1.3.0 (Aramaki et al., 2020), which employs HMMER searches against the KEGG Orthology (KO) database (Kanehisa and Goto, 2000). Based on KO annotations, genes associated with carbon, nitrogen, and sulfur cycling, as well as genes potentially involved in microbial adaptation to high-salinity stress, were specifically extracted. The relative abundance of each KO was calculated by summing the CPM-normalized abundances of all genes annotated to that KO.

The MAG reconstruction, reads were reassembled using the assembly module of the MetaWRAP v1.2.1 pipeline (Uritskiy et al., 2018), which internally uses MEGAHIT v1.1.3 (Li et al., 2016). To enhance the reliability of downstream genome binning, contigs shorter than 2,000 bp were removed. Assembly quality metrics were evaluated using QUAST v5.0.2 (Gurevich et al., 2013). MAG reconstruction was performed using the binning module of MetaWRAP, which integrates three independent binning algorithms: MetaBAT2 v2.15 (Kang et al., 2019), MaxBin2 v2.2.7 (Wu et al., 2016), and CONCOCT v1.1.0 (Alneberg et al., 2014). The resulting bins were subsequently refined using the bin_refinement module in MetaWRAP. The completeness and contamination levels of all refined bins were assessed with CheckM v1.1.3 (Parks et al., 2015), and only bins exhibiting ≥75% completeness and ≤10% contamination was retained for downstream analyses. High-quality MAGs were dereplicated using dRep v3.4.2 (Olm et al., 2017) employing a two-step clustering strategy. Primary clustering involved MASH-based pre-screening using a ≥ 95% average nucleotide identity (ANI) threshold and a minimum genome alignment fraction (−nc 0.3) of ≥30%. Secondary clustering was conducted at the species level using a ≥ 95% ANI threshold over ≥30% aligned genomic regions (−sa 0.95), ensuring high-resolution dereplication. The relative abundance of each dereplicated MAG across samples was calculated using CoverM v0.7.0,^2^ with parameters --min-read-percent-identity 0.95 and --min-covered-fraction 0.75.

### 2.4 Statistical analyses of microbial diversity

Redundancy Analysis (RDA) was applied to explore the relationships between environmental factors and microbial community composition at the phylum level. Environmental variables included temperature (Temp), salinity, total organic carbon (TOC), and total nitrogen (TN). RDA analyses were conducted using the vegan package in R (v4.2.3).

### 2.5 Taxonomy and functional annotation

The taxonomy of dereplicated MAGs was determined using GTDB-Tk v2.2.6 (Chaumeil et al., 2020), a standardized toolkit for prokaryotic genome classification, with reference to the GTDB R214 database. To validate taxonomic assignments, a maximum-likelihood phylogenetic tree was constructed using pplacer v1.1 (Matsen et al., 2010), aligning MAGs to the GTDB-Tk reference tree. Taxonomic assignments were further cross-validated through marker gene phylogenies and Average Nucleotide Identity (ANI) clustering. Phylogenetic trees were visualized using the ChiPlot online platform.^3^ Functional annotation of MAGs was performed using PROKKA v1.14.5 (Seemann et al., 2014) for standardized gene calling and functional assignment. KEGG Orthology (KO) annotations were assigned using KofamScan v1.3.0 (Aramaki et al., 2020) with a minimum score threshold of 0.7. Pathway completeness for each MAG was evaluated using KEGG Decoder, with a focus on biogeochemical processes, including carbon, nitrogen, and sulfur metabolism. In addition, METABOLIC v4.0 (Zhou et al., 2022) was used to predict metabolic potentials and biogeochemical functions across MAGs, providing high-resolution insights into microbial ecological traits.

### 2.6 Data availability

The original research findings from the study are openly accessible to the public. The dataset is located at the following link: https://www.ncbi.nlm.nih.gov/bioproject/PRJNA1200918/.

## 3 Results

### 3.1 Study site and sample collection

**Figure 1:**
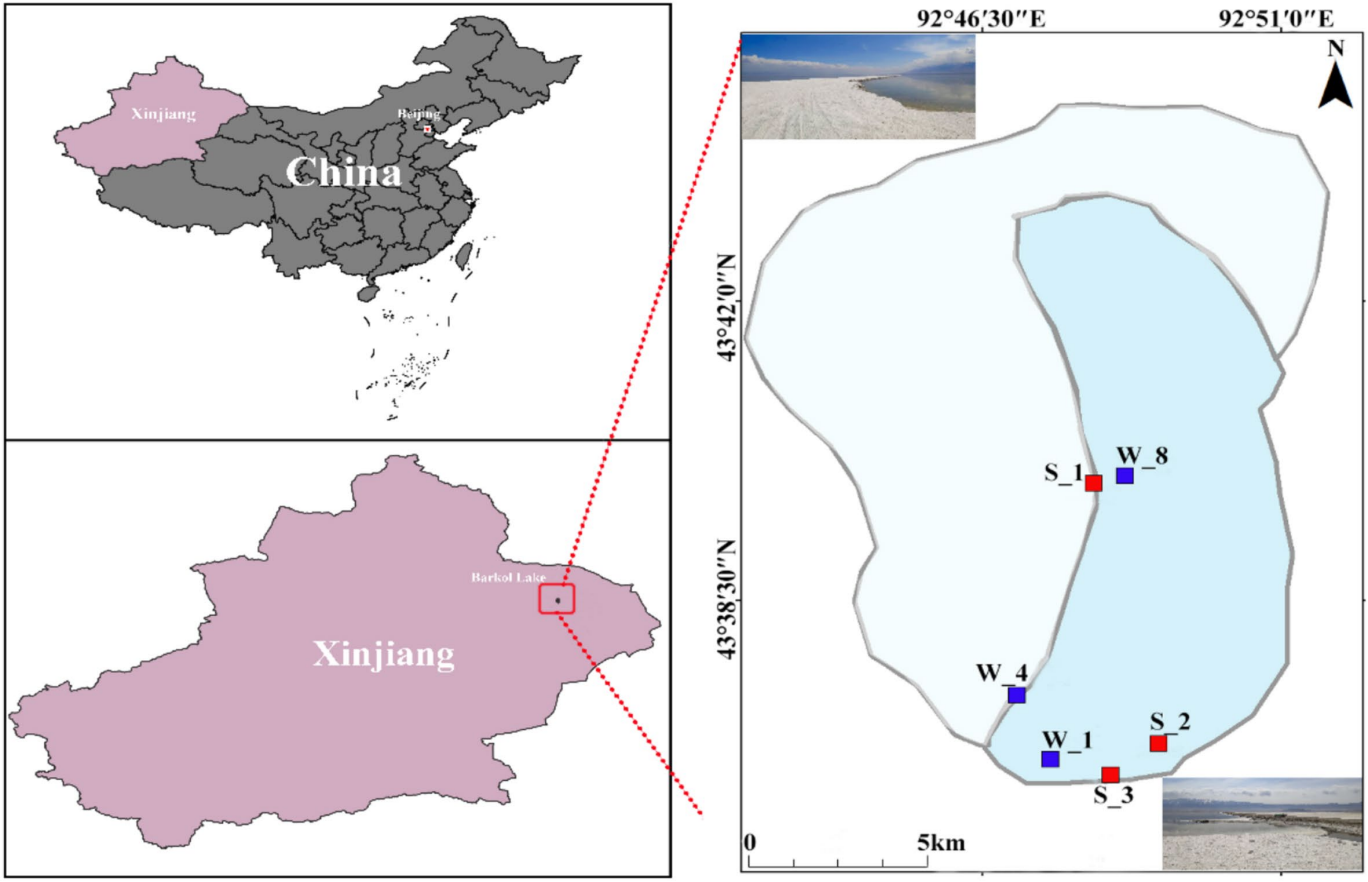
Location of sampling sites in Lake Barkol, Xinjiang Province, China. The maps show the geographic position of Lake Barkol within China and Xinjiang Province. The right panel presents the detailed distribution of sampling sites around the lake, where red squares indicate sediment sampling locations and blue squares indicate lake water sampling locations. Insets provide photographs highlighting the lake’s typical salt flat environment and surrounding landscape. The base map was adapted from Tianditu (https://www.tianditu.gov.cn/), the National Platform for Common Geospatial Information Services of China, hosted by the Ministry of Natural Resources of the People’s Republic of China.

Six samples were collected from Lake Barkol in August 2019 and November 2020, comprising three surface water samples (W_1, W_4, W_8) and three sediment samples (S_1, S_2, S_3) (Figure 1). Environmental parameters exhibited marked variation between sample types and across seasons (Table 1). Salinity ranged from 9.5 to 20.2%, while pH values remained relatively stable, ranging from 7.99 to 8.18. Total organic carbon (TOC) and total nitrogen (TN) concentrations were consistently higher in water samples (TOC: 85–185 mg/L; TN: 16–34 mg/L) compared to sediment samples (TOC: 33–67 mg/L; TN: 11–29 mg/L). Temperature differences were also pronounced between seasons, with summer water samples ranging from 19.7°C to 29.9°C, whereas sediment samples collected in late autumn showed significantly lower temperatures (0.2–2.4°C). These gradients indicate clear seasonal and habitat-based environmental differentiation across Lake Barkol.

**Table 1:** Sample information and environmental characteristics of lake water and sediment samples from Lake Barkol.

Sample	Sampling date	Sample type	GPS	Temp (°C)	pH	Salinity (%)	Cond. (SPC) μS/cm	TOC (mg/L)	TN (mg/L)
W_1	Aug-19	Brine	43°36′39″ N 92°47′29″E	29.9	8.18	15.4	125,166	90	19
W_4	Aug-19	Brine	43°37′24″ N 92°47′1″E	21.6	8.02	20.2	151,020	185	34
W_8	Aug-19	Brine	43°39′59″ N 92°48′37″E	19.7	8.11	15.9	121,327	85	16
S_1	Nov-20	Sediment	43°41′01″ N 92°47′50″E	0.2	8.00	15	ND	67	29
S_2	Nov-20	Sediment	43°36′52″ N 92°49′06″E	1.9	7.99	11.5	ND	54	21
S_3	Nov-20	Sediment	43°36′36″ N 92°47′29″E	2.4	8.00	9.5	ND	33	11

### 3.2 Metagenomic zonation of carbon, nitrogen, and sulfur cycling pathways and stress responses in hypersaline Lake Barkol

Gene-centric metagenomic analysis of Lake Barkol, a hypersaline inland lake, revealed spatial partitioning of carbon (C), nitrogen (N), and sulfur (S) cycling pathways between the oxygenated water column (W1-W8) and anoxic sediments (S1-S3), driven by salinity gradients and redox stratification. From 12.48 million predicted open reading frames (ORFs), identified 8.1 million non-redundant ORFs, among which 6,278 unique KO identifiers were annotated, reflecting a wide range of metabolic functions. To quantify pathway-level activity, CPM (Counts Per Million) values were calculated for each KO across samples, enabling comparative analysis of carbon, nitrogen, and sulfur cycling gene abundances between water and sediment environments (Supplementary Table S1a).

Carbon cycling genes showed stratified distribution. Reductive CO₂ fixation pathways via the WL pathway were enriched in sediments (cdhD, cdhE, cooS; cooS: S2 = 445.4 vs. W1 = 6.0), along with methanogenesis genes (mttB: S2 = 1888.9 vs. W1 = 341.8). In the water column, phototrophic carbon fixation genes (rbcL: S1 = 76.7 vs. W1 = 7.3) and fermentation-related genes (fdh: W4 = 837.1) were more abundant. Reverse TCA cycle activity was low (aclA: S2 = 2.3). Methane oxidation genes (pmoB, mmoX) were not detected. Anoxygenic photosynthesis gene pufM was present at low levels in the water column (W1 = 43.9) (Supplementary Figure S1). Nitrogen cycling genes also demonstrated spatial separation. Sediments exhibited higher abundances of genes for denitrification (norB: S1 = 41.2 vs. W1 = 33.3), dissimilatory nitrate reduction to ammonium (DNRA; nrfA: S1 = 91.8 vs. W1 = 12.7), and ammonification (gdhA: S1 = 375.2 vs. W1 = 91.4). Water column samples contained more genes for assimilatory nitrate reduction (nasA: W8 = 15.6 vs. S1 = 0.0) and nitrogen fixation (nifD: W1 = 1.9 vs. S1 = 15.3). Ammonium transporter genes (amt) were abundant in both environments (W1 = 367.1; S1 = 469.7). Genes associated with nitrification (hao: S1 = 67.3) and anammox were of low abundance or undetected (Supplementary Figure S2). Sulfur cycling gene profiles exhibited similar compartmentalization. Sediments showed elevated dissimilatory sulfate reduction genes (aprA: S1 = 122.0 vs. W1 = 6.2; dsrA: S1 = 41.4), and water column samples had higher abundances of tst (W1 = 371.5 vs. S1 = 233.5). Aerobic sulfide oxidation genes (sqr: W4 = 318.1) were abundant in both layers. Thiosulfate oxidation genes (soxB: S1 = 162.7 vs. W1 = 129.5) and assimilatory sulfate reduction genes (cysH, cysN) were more abundant in sediments (Supplementary Figure S3).

Salinity adaptation genes were differentially enriched. Sediment metagenomes contained higher abundances of Na^+^/H^+^ antiporter genes (mnhD: S1 = 838.7 vs. W1 = 651.3) and compatible solute synthesis genes (ectA: S1 = 77.8; betA: S1 = 133.1). Water column samples showed higher expression of K^+^ transporters (ktrB: W4 = 1116.6) and trehalose biosynthesis genes (otsB: S2 = 305.9). Ectoine degradation genes (doeD: S1 = 75.2 vs. W1 = 184.9) were detected in both. Stress response genes showed compartment-specific patterns. Water column samples contained higher DNA repair gene abundance (uvrA: W1 = 958.0), whereas sediment samples contained more halorhodopsin (hop: S1 = 5.9). Osmoprotectant transporter genes (proX) were more abundant in sediments (S1 = 551.2 vs. W1 = 287.7). Mechanosensitive channel genes (mscS) were detected in the water column (W1 = 320.6). Redox- and salinity-associated metabolic gene profiles defined distinct microbial niches in Lake Barkol. Sediments were characterized by enrichment of genes involved in reductive carbon, nitrogen, and sulfur pathways, coupled with osmoprotectant biosynthesis and ion transport. Water column samples exhibited greater abundance of genes related to oxidative metabolism, DNA repair, and osmotic regulation. These patterns delineate stratified functional potential of microbial communities under hypersaline, redox-structured conditions (Supplementary Figure S4).

### 3.3 Taxonomic composition of MAGs

**Figure 2:**
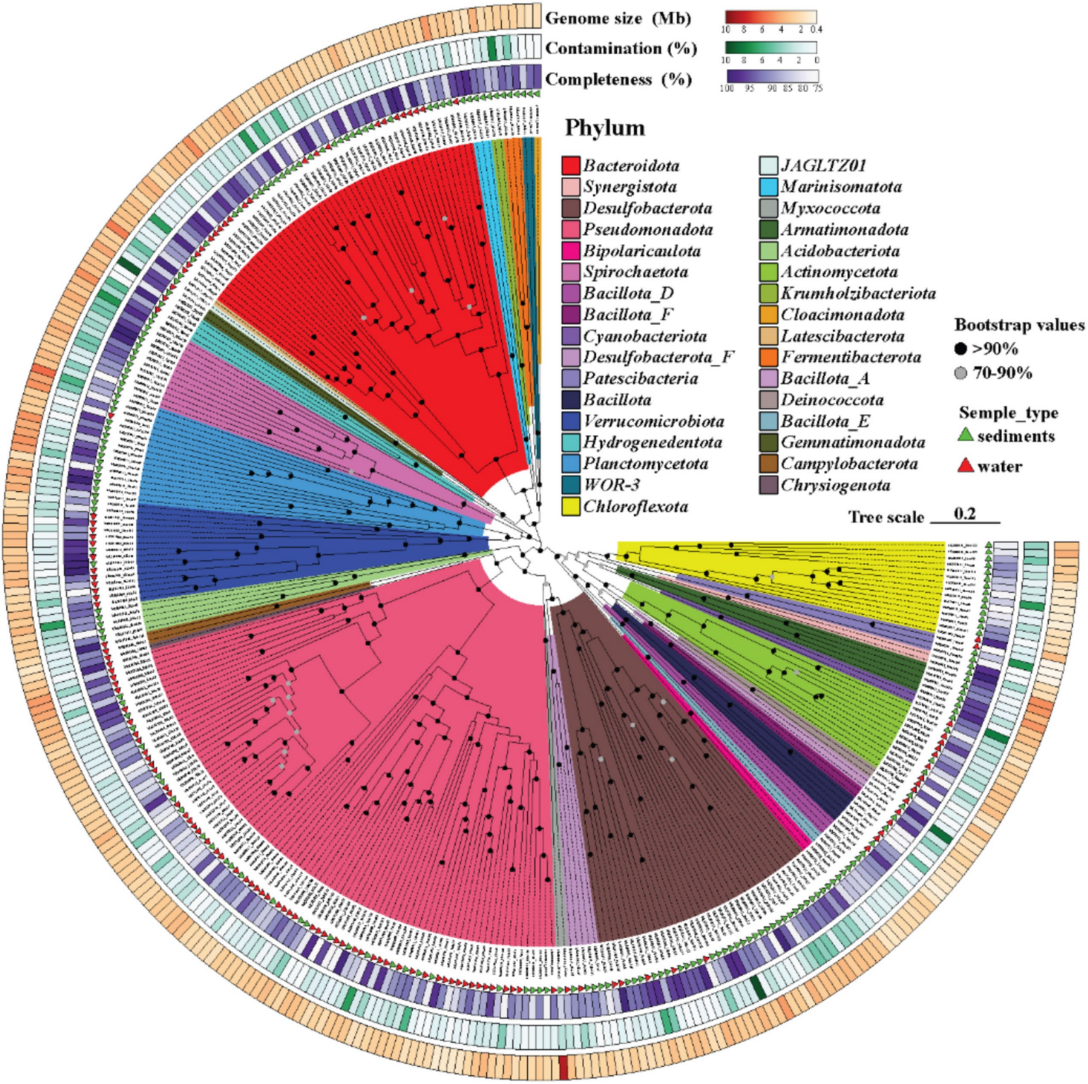
Microbial diversity in Lake Barkol water and sediment. A. Phylogenetic relationships among the bacterial MAGs recovered from the sediment and water of Lake Barkol were inferred from a multiple sequence alignment of 120 bacterial marker proteins, as defined by the Genome Taxonomy Database classifier (GTDB-Tk). The colors of the branches represent different phyla.

**Figure 3:**
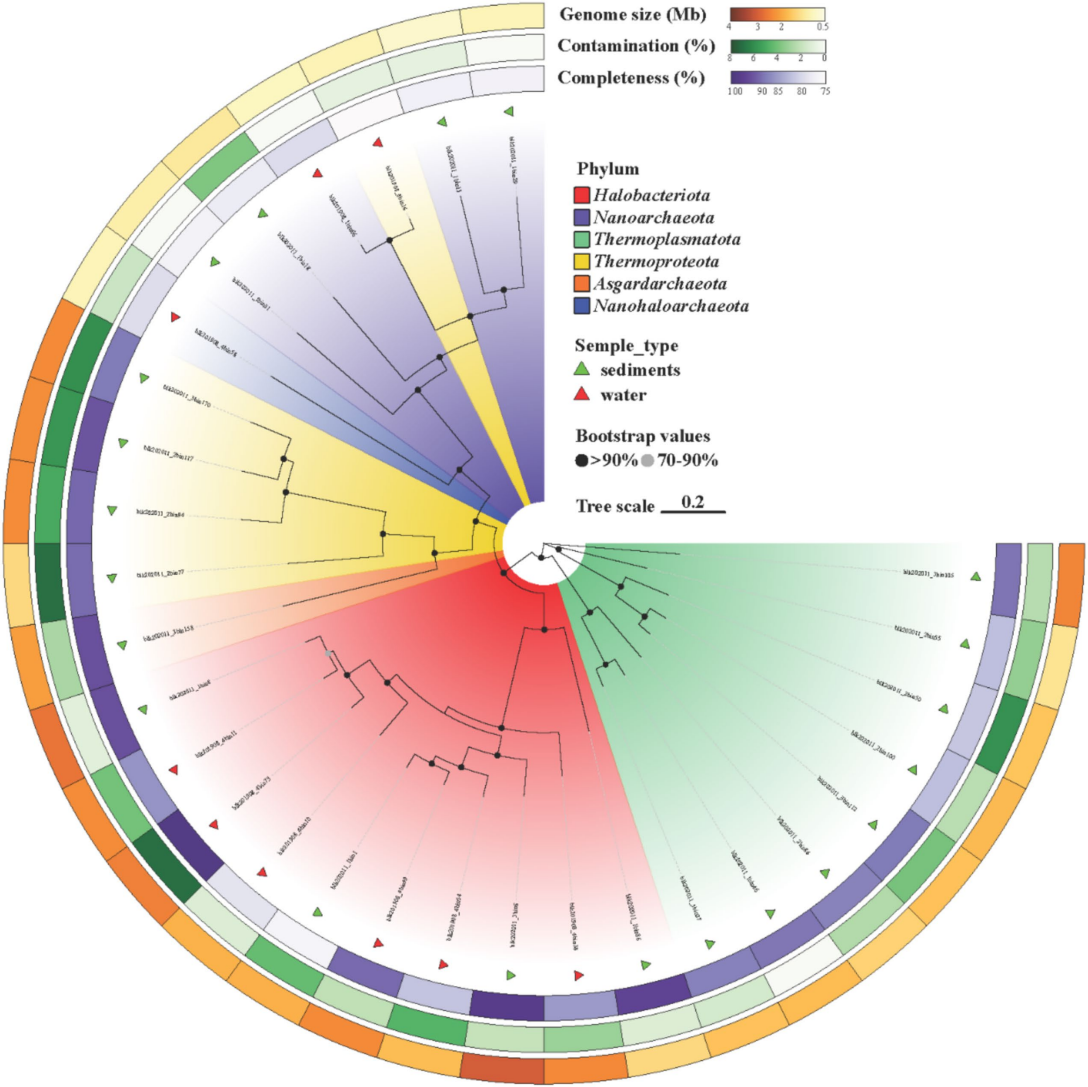
Phylogenetic relationships among the 30 archaeal MAGs were inferred by constructing a maximum-likelihood tree using 122 archaeal marker genes identified by GTDB-Tk. Concatenated multiple sequence alignment was performed using the GTDB-Tk alignment module. Branch colors represent different phyla.

**Figure 4:**
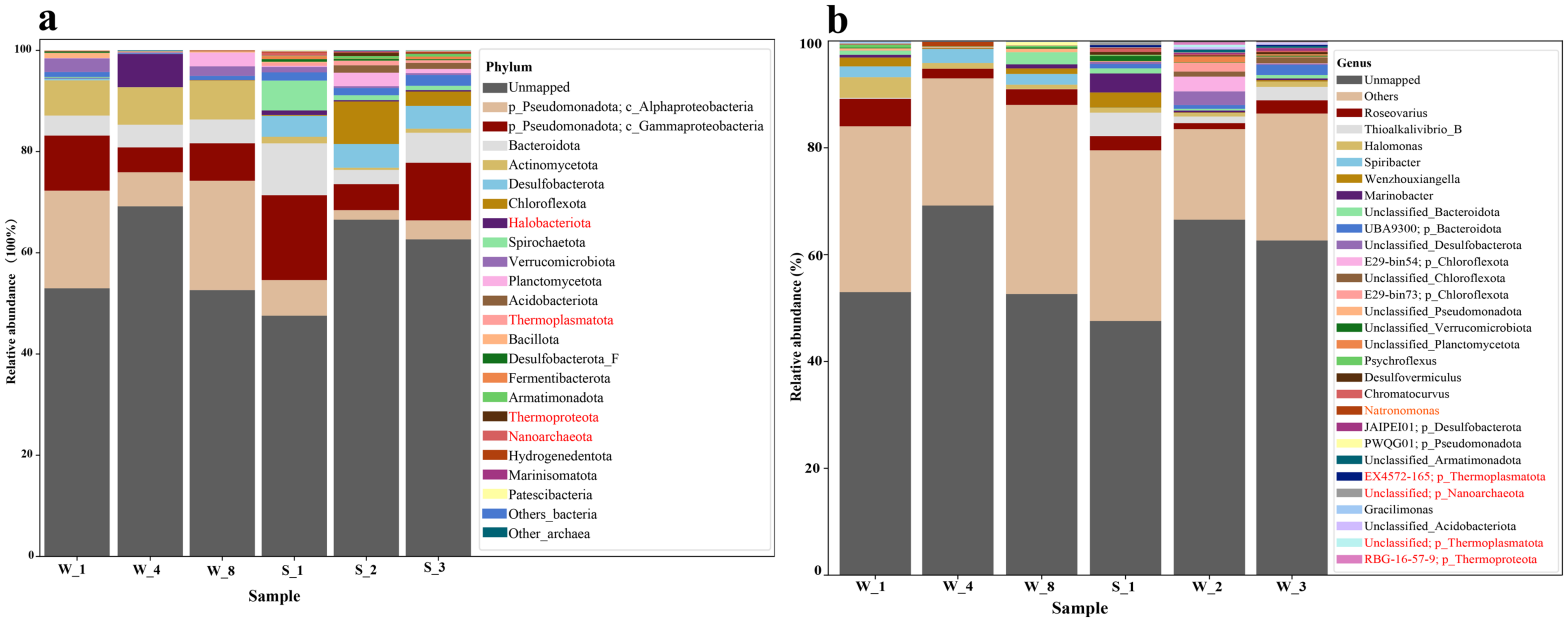
Taxonomic composition of microbial communities in water (W_1, W_4, W_8) and sediment (S_1, S_2, S_3) samples from Lake Barkol. **(A)** Relative abundance of the top 20 microbial phyla. Archaeal phyla are highlighted in red. Phyla outside the top 20 are grouped as “Other.” “Unmapped” represents sequences that could not be taxonomically assigned at the phylum level. **(B)** Relative abundance of the top 30 microbial genera. Archaeal genera are highlighted in red. Genera outside the top 30 are grouped as “Other”.

Sequencing and assembly of water and sediment samples generated 90,686–85,871 and 146,935–208,102 contigs, respectively, with water-derived contigs exhibiting higher assembly quality, including longer average lengths and N50 values (7,114–10,810 bp vs. 5,559–6,485 bp in sediments) (Supplementary Table S1b). From these, 309 non-redundant metagenome-assembled genomes (MAGs) were reconstructed under stringent criteria (ANI ≥ 95%, completeness ≥ 75%, contamination ≤ 10%), of which 168 met high-quality standards (completeness ≥ 90%, contamination ≤ 5%). These MAGs spanned a size range of 0.49 to 8.57 Mb with an average N50 of 23,644 bp and exhibited a broad GC content variation 25.3–75.3% (Figure 2; Supplementary Table S3). Taxonomic classification resolved 33 bacterial phyla, dominated by *Pseudomonadota* (*Gammaproteobacteria*: 51 MAGs; *Alphaproteobacteria*: 37 MAGs), *Bacteroidota* (50 MAGs), and *Desulfobacterota* (37 MAGs), alongside *Planctomycetota*, *Verrucomicrobiota*, and *Actinobacteriota*. Archaeal MAGs (*n* = 30) were primarily affiliated with *Halobacteriota* (10 MAGs) and *Thermoplasmatota* (8 MAGs), consistent with adaptations to hypersaline conditions. Sediment samples consistently showed higher microbial abundance (S_1: 52.42%) compared to water samples (W_4: 30.81%) (Figure 3A), correlating with lower salinity (9.5–15%) and temperature (0.2–2.4°C) in sediments. In contrast, elevated salinity (20.2%) and temperature (21.6°C) in brine samples restricted diversity, highlighting these factors as key environmental filters. Redundancy Analysis (RDA) (Supplementary Figure S5) further demonstrated that water communities (W_8) aligned with temperature and salinity gradients, supporting the dominance of halophilic taxa such as *Halobacteria* (*Halobacteriota*) and *Wenzhouxiangellaceae*, whereas sediment communities (S_1) correlated with total organic carbon (TOC) and total nitrogen (TN), reflecting the prevalence of taxa like the unclassified *Dehalococcoidia* lineage *AB-539-J10* and *Pontimonas* (*Actinobacteriota*), which reached 22.59% relative abundance in S_1. *Pontimonas’s* association with moderate salinity (9.2–15.4%) and seasonal dynamics (Gorrasi et al., 2022) aligns with its role in organic matter utilization under varying conditions (Figure 3B). Archaeal communities diverged sharply, with water dominated by *Halobacteria* and sediments enriched in *Thermoplasmatota* lineages (*EX4484-6* and *E2*), known for low-energy habitat adaptations (Pavloudi and Zafeiropoulos, 2022). Twelve archaeal MAGs remained unclassified at the genus level, underscoring the prevalence of uncultivated lineages (Figure 4; Supplementary Table S3). These results illustrate how temperature, salinity, and nutrient availability drive microbial niche partitioning, shaping taxonomic composition and biogeochemical cycling in Lake Barkol’s hypersaline ecosystem.

### 3.4 Diversity of carbon metabolic pathways and major microbial contributors in Lake Barkol

**Figure 5:**
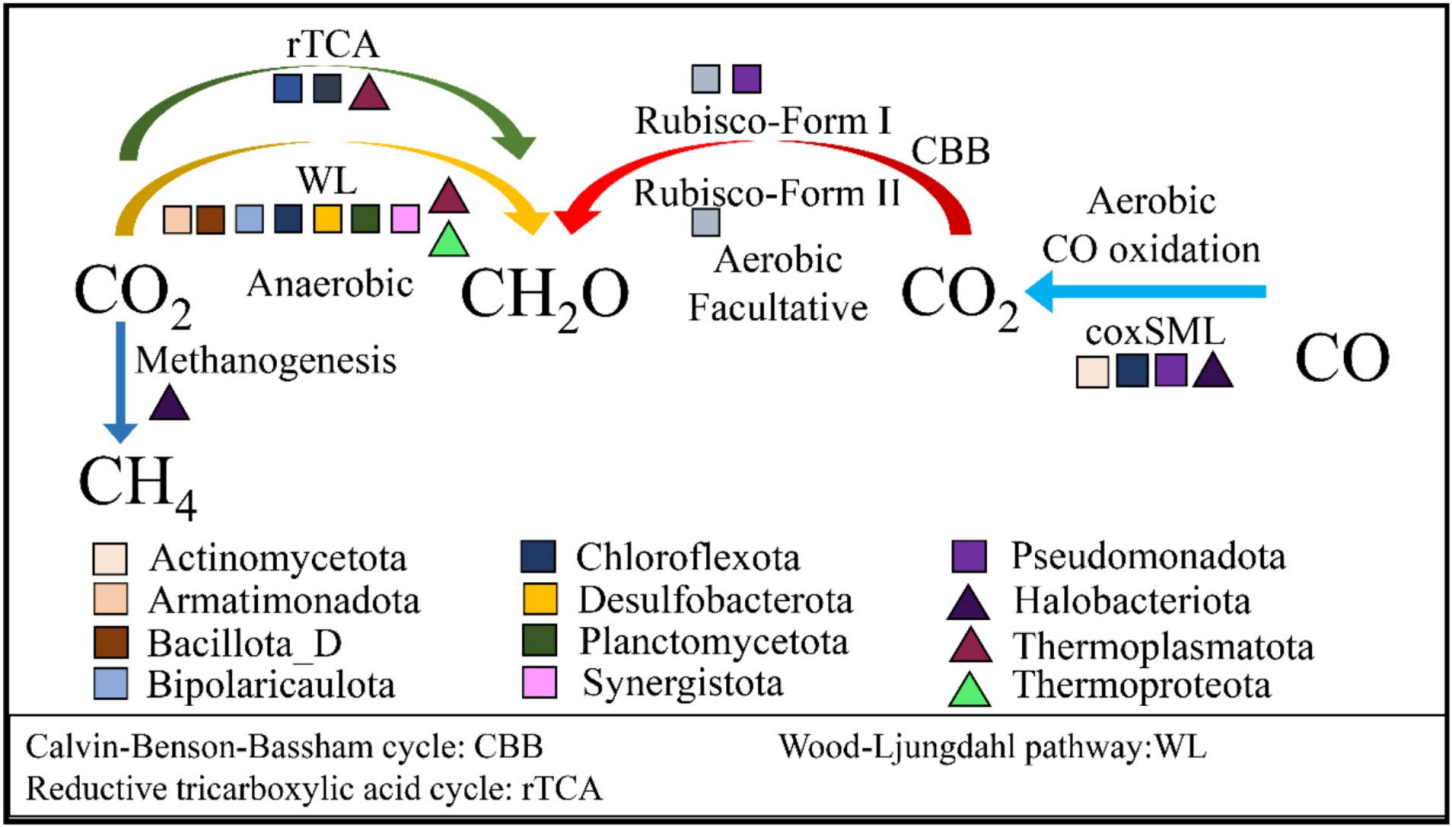
Functional profiles of the main microbial groups involved in main carbon cycling in Lake Barkol. The red arrow is the Calvin-Benson-Bassham (CBB) cycle, the yellow arrow is the rTCA cycle, the green arrow is Wood-Ljungdahl (WL) pathway, the light blue arrow is Aerobic CO oxidation pathway, and the blue arrow is methanogenesis pathway. The square is bacteria phyla, and the triangle is archaea phyla. Complete lists of metabolic genes or pathways can be found in Supplementary Table S4.

The carbon cycle is crucial for the primary productivity and stability of ecosystems. Lake Barkol harbors diverse carbon fixation and organic carbon transformation pathways, reflecting the metabolic versatility and functional redundancy of its microbial communities under extreme hypersaline conditions (Figure 5; Supplementary Tables S4, S5). By integrating MAG based functional annotation with gene abundance profiling, we identified distinct microbial phyla that contribute differentially to major carbon metabolism routes across sediment and water environments.

The CBB cycle is autotrophs convert inorganic carbon into organic carbon, playing a central role in primary production (Mobberley et al., 2013; Ebenhöh and Spelberg et al., 2018), and is the most widespread carbon fixation pathway on Earth, commonly found in cyanobacteria, plants, algae, and certain chemotrophic bacteria. In Lake Barkol, predominantly associated with *Cyanobacteriota* and *Pseudomonadota*, with gene expression almost exclusively observed in water column samples. This distribution underscores the reliance of the CBB cycle on phototrophic organisms and light-penetrating aquatic habitats. The key enzyme ribulose-1,5-bisphosphate carboxylase/oxygenase (RuBisCO) was detected in multiple MAGs, with both Form I and Form II RuBisCO genes present in one MAG associated with *Hydrogenobacteria*, while other MAGs contained only Form I genes. These findings suggest the potential for autotrophic metabolism via the CBB cycle in these microbes, though further validation via transcriptomics or stable isotope labeling is necessary. Metagenomic analyses *Gammaproteobacteria* encoding a complete set of CBB cycle genes, supporting their putative role in carbon fixation under high-salinity conditions (Vavourakis et al., 2016). This aligns with previous studies demonstrating that *Gammaproteobacteria* contribute significantly to primary production in high-salinity lake sediments and Tibetan Plateau saline-alkaline lakes (Liu et al., 2018). Specifically, autotrophic extremophiles (e.g., *Ectothiorhodospiraceae*) and chemolithoautotrophic lineages (e.g., *Thioalkalivibrio*) have been shown to play pivotal roles in carbon cycling (Fang et al., 2022; Kovaleva et al., 2011). Our findings further support the hypothesis that these microbial groups possess autotrophic capabilities via the CBB cycle, emphasizing the ecological significance of *Gammaproteobacteria* in saline environments. Additionally, two MAGs assigned to Cyanobacteria (family *Rubidibacteraceae* and *Cyanobiaceae*) were detected, both known halophiles capable of thriving at salt concentrations exceeding 100–150 g/L (Oren, 2011). These MAGs likely contribute to carbon fixation via photosynthesis, supporting primary production in Lake Barkol.

The WL pathway is a predominant carbon fixation mechanism in anaerobic microbes, allowing CO₂ conversion into acetyl-CoA, a central metabolic intermediate. This pathway operates with extremely low ATP requirements (<1 ATP per cycle) and is well-adapted to low-energy environments (Bar-Even et al., 2012). In Lake Barkol is primarily active in sediment samples and 48 MAGs associated with the WL pathway were identified, is largely contributed by *Desulfobacterota*, *Chloroflexota*, *Planctomycetota*, *Armatimonadota*, *Bacillota_D*, *Bipolaricaulota*, and *Spirochaetota*, as well as archaeal phyla *Thermoproteota* and *Thermoplasmatota*. The dominance of this low-energy-demanding pathway in anoxic environments supports its ecological advantage under energy-limited and these microbes play a major role in anaerobic carbon cycling, particularly under highly saline and reducing conditions. Additionally, some microorganisms fix carbon through the rTCA cycle, commonly found in autotrophic and anaerobic photoautotrophic bacteria in anaerobic or microaerobic environments (Berg, 2011; Bhatnagar et al., 2020). The rTCA cycle, as an alternative to the WL pathway, provides essential intermediates for energy metabolism and appears to be particularly well-adapted to high-temperature and low-oxygen environments. Several studies have demonstrated that the rTCA cycle is one of the dominant carbon fixation pathways in hydrothermal vents (Hügler and Sievert, 2011). Moreover, the rTCA cycle is prevalent in non-saline soils on the Qinghai-Tibet Plateau (Li et al., 2021). In our study, rTCA cycle is driven mainly by *Chloroflexota*, *Desulfobacterota*, and the *Sulfurovaceae*, family of *Campylobacterota* are chemolithoautotrophs that oxidize reduced sulfur compounds and molecular hydrogen, their metabolic versatility allows adaptation to diverse ecological niches, including deep-sea hydrothermal vents and brine-seawater interfaces (Alamoudi et al., 2025). Additionally, 4 MAGs from *Thermoplasmatota*, which encode the rTCA pathway, were obtained from sediment samples. The broader ecological niche of the rTCA cycle indicates that these representative microbial groups and their physiological adaptations play a key role in adapting to extreme saline environments.

Genes involved in methanogenesis are predominantly encoded by *Halobacteriota*. A *Methanohalophilus halophilus* MAG (95.58% completeness, 1.31% contamination) was identified in sediment samples. This taxon is a known halophilic methanogen, capable of metabolizing substrates such as methanol, acetic acid, dimethylsulfide, trimethylamine, and dimethylamine. To cope with osmotic stress, *M. halophilus* synthesizes organic solutes, which also serve as substrates for methane metabolism (Garrity and Stanley, 2001).

In aerobic environments, carbon monoxide (CO) oxidation is mediated by type I carbon monoxide dehydrogenase (CODH), which channels electrons into either ATP generation or the CBB cycle for CO₂ fixation (King and Weber, 2007; Bay et al., 2021). We detected 40 MAGs encoding the coxSML gene cluster associated with CO oxidation, spanning *Pseudomonadota* (*Alpha*- and *Gammaproteobacteria*), *Actinomycetota*, *Chloroflexota*, and *Halobacteriota* (genus *Natronomonas*). These microbes play a key role in CO detoxification and serve as an alternative energy source under organic carbon limitation (Cordero et al., 2019; Leung et al., 2024). Fermentative metabolism is broadly distributed and contributed by multiple phyla including *Pseudomonadota*, *Bacteroidota*, and *Chloroflexota*, consistent with widespread anaerobic degradation of organic matter. These results demonstrate a clear ecological partitioning of carbon metabolism in Lake Barkol, phototrophic carbon fixation via the CBB cycle dominates the water column, while anaerobic autotrophy via the WL and rTCA pathways prevails in sediments. This functional differentiation highlights the adaptation of microbial communities to the lake’s redox gradients and extreme salinity, underpinning primary productivity and biogeochemical resilience in this hypersaline ecosystem.

### 3.5 Nitrogen metabolism in Lake Barkol

**Figure 6:**
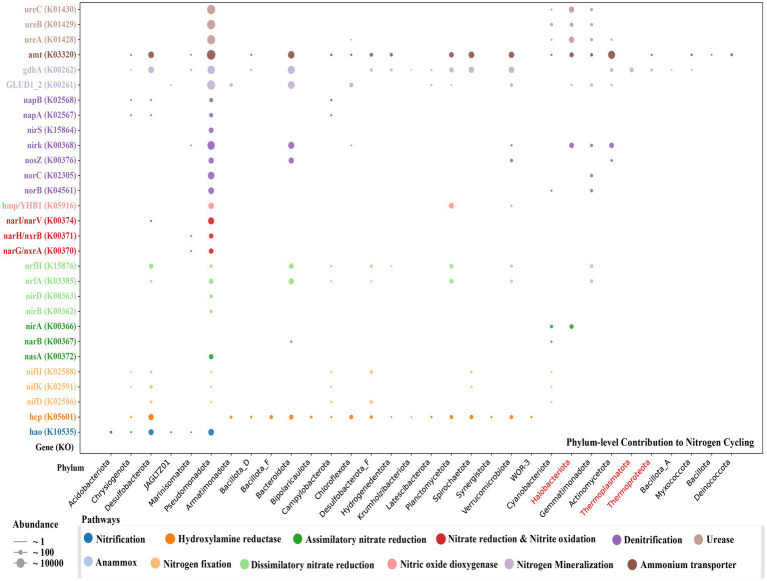
Phylum-level distribution and relative abundance of key nitrogen cycling genes (KEGG Orthology identifiers) in microbial communities from Lake Barkol. Dot size represents gene abundance, and color indicates functional category. Archaeal phyla are shown in red.

The nitrogen cycle in Lake Barkol is strongly structured by environmental gradients between the oxygenated water column and the anoxic sediment, as reflected by the spatial distribution of functional genes and metabolic pathways. Analysis of KEGG ortholog abundance revealed that nitrogen fixation, nitrification, ammonification, dissimilatory nitrate reduction (DNRA) pathways were more enriched in sediment, while urease activity, ammonium transport and assimilatory nitrate reduction dominated in the water column (Figure 6; Supplementary Tables S4, S5).

Microorganisms convert atmospheric nitrogen into ammonia through the nitrogen fixation pathway (nifD, nifH, nifK), ensuring a continuous source of nitrogen to the lake ecosystem. The nifH gene binds the iron–sulfur cofactor, whereas nifD and nifK catalyze key steps in nitrogenase activity (Gruber and Galloway, 2008). In this study, nitrogen fixation genes were identified in multiple taxonomic groups, including *Campylobacterota*, *Cyanobacteriota, Desulfobacterota*, and *Pseudomonadota*.

A total of 15 MAGs harbored napAB or narGHI genes for nitrate reduction, while 44 MAGs carried nrfAH and nirBD, completing the DNRA pathway. These were affiliated with *Campylobacterota*, *Chrysiogenota*, *Desulfobacterota*, *Marinisomatota*, and *Pseudomonadota*, underscoring DNRA’s ecological role in nitrogen retention. Denitrification, a key nitrogen loss pathway, converts nitrate to N₂. We identified nirK and nirS genes in five bacterial and one archaeal MAG. Additionally, *Pseudomonadota* and *Gemmatimonadota* MAGs encoded norBC genes, catalyzing NO to N₂O reduction. While nitrate may originate from external inputs or *in situ* processes, the apparent absence of *amoABC* genes suggests that canonical nitrification is incomplete or suppressed in Lake Barkol.

The urea hydrolysis pathway (ureABC) was present in 51 MAGs, affiliated with *Pseudomonadota*, *Gemmatimonadota*, *Cyanobacteriota*, and *Halobacteriota*, providing an additional nitrogen source. Ammonia released via urea degradation supports microbial growth, while concomitant CO₂ production may benefit phototrophs (Gruber, 2008). Collectively, these findings reveal a nitrogen cycle shaped by functional redundancy and metabolic plasticity, where canonical pathways like nitrification may be suppressed by hypersaline and redox stressors (Bernhard et al., 2005; Boey et al., 2022), and microbial communities compensate via alternative routes to maintain nitrogen flux and ecosystem stability.

### 3.6 The main sulfur cycling processes dominated by sulfur-oxidizing and sulfur-reducing microbes in Lake Barkol

**Figure 7:**
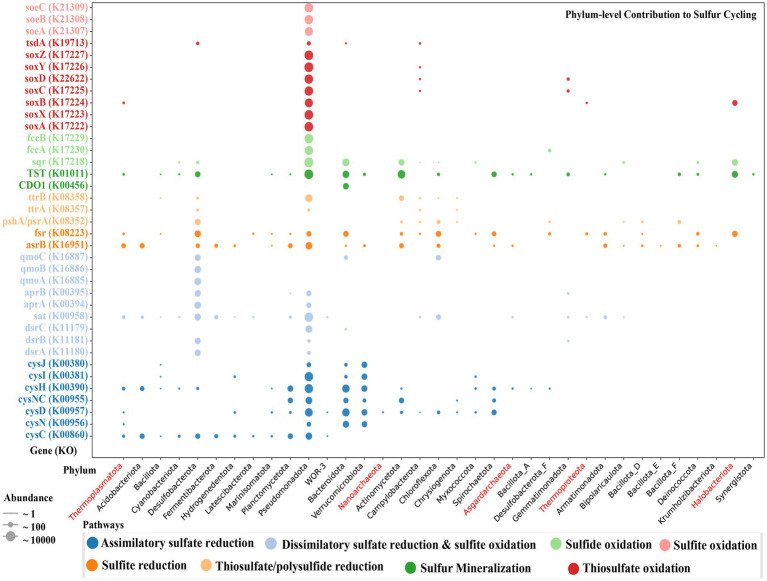
Phylum-level distribution and relative abundance of key sulfur cycling genes (KEGG Orthology identifiers) in microbial communities from Lake Barkol. Dot size represents gene abundance, and color indicates sulfur metabolic pathway. Archaeal phyla are shown in red.

The biogeochemical sulfur cycle is a key driver of microbial redox processes in athalassohaline lake ecosystems, where salinity and stratified redox conditions shape microbial metabolism (Holmer and Storkholm, 2001; Oren, 2011). Sulfur oxidation and reduction are the main metabolic processes in Lake Barkol. Sulfur-oxidizing bacteria (SOBs) are widely distributed in hypersaline and alkaline ecosystems, particularly in neutral-pH, high-salinity environments such as inland salt lakes and salt flats, where they exhibit substantial taxonomic and metabolic diversity (Sorokin et al., 2006). In this study, we reconstructed 14 high-quality MAGs affiliated with the families *Rhodobacteraceae*, *Halothiobacillaceae*, and *Thiomicrospiraceae*, all of which harbor genes encoding a complete Sox enzyme complex (*soxXYZABCD*), indicating potential for thiosulfate oxidation. Genes encoding SoxA/XYZ components were detected primarily in *Gammaproteobacteria*, including members of the *Thioalkalivibrionaceae*, *Thiohalospiraceae*, and unclassified UBA6429. Sulfide:quinone oxidoreductase (sqr) genes were present in members of *Pseudomonadota*, *Campylobacterota*, and *Cyanobacteriota*. Additionally, fccA and fccB, encoding flavocytochrome c sulfide dehydrogenase, were found in *Thioalkalivibrionaceae* MAGs, suggesting active sulfide oxidation under saline-alkaline conditions. These results are consistent with observations from global hypersaline environments, such as soda lakes, salt marshes, and deep saline pools, where SOBs exhibit conserved sulfur metabolic strategies (Wood and Kelly, 1991; Giri et al., 2004; McGonigle et al., 2022; Qiu et al., 2024). The SOBs identified in Lake Barkol thus exemplify the metabolic convergence driven by hypersaline selective pressures and underscore their ecological significance in sulfur turnover under extreme conditions (Figure 7; Supplementary Tables S4, S5).

Sulfate, a key intermediate in the sulfur cycle, is commonly produced via microbial-mediated sulfide oxidation. Sulfate-reducing microorganisms (SRMs) utilize sulfate as a terminal electron acceptor during anaerobic respiration. While most SRMs thrive under neutral to slightly alkaline conditions, some exhibit remarkable tolerance to extreme environments, including hot springs and deep-sea hydrothermal vents (Muyzer and Stams, 2008). Hypersaline environments such as salt lakes, marshes, and pans serve as important habitats for SRMs that maintain sulfate-reducing activity despite high osmotic stress (Oren, 2011). SRMs share the same sulfate respiration pathway, and their core enzymes include ATP sulfurylase, adenosine sulfate reductase, and dissimilatory sulfite reductase. In this study, we identified 16 MAGs affiliated with *Desulfobacterota* encoding the complete dissimilatory sulfate reduction (dsr) pathway, suggesting their role in reducing sulfate to sulfide. Previous studies on Lake Barkol have shown that *Desulfobacterota* may represent a key microbial group involved in the lake’s biogeochemical cycles, particularly in the sulfur cycle and its coupling with other biogeochemical processes (Liu et al., 2024). The reverse dissimilatory sulfate reduction (rDsr) pathway, previously observed in phototrophic and chemolithotrophic sulfur-oxidizing microorganisms (SOMs), was also detected in our metagenomic data (Jia et al., 2025). Specifically, chemolithoautotrophic genera such as *Thiohalophilus* and *Thioalkalivibrio_B* were found to harbor *sat*, aprAB, dsrAB, and soxA/XYZ, indicating potential for sulfide oxidation via the rDsr pathway. These organisms may also contribute to carbon fixation through the CBB cycle.

A total of 161 MAGs harbored at least one gene associated with assimilatory sulfate reduction, including cycND, cysC, cysH, and cysJI. Notably, five MAGs were affiliated with archaeal lineages, including *Thermoplasmatota* (class E2) and *Nanoarchaeota* (order *Woesearchaeales*), expanding the known taxonomic diversity of sulfate-assimilating organisms in hypersaline ecosystems. This process likely relies on syntrophic or complementary metabolic interactions among diverse microbial taxa that facilitate sulfate assimilation in distinct ecological niches. The sulfur cycle in Lake Barkol is mediated by phylogenetically diverse communities, primarily *Pseudomonadota* and *Desulfobacterota*, along with archaeal taxa, extending the known lineages contributing to sulfur metabolism (Figure 7). These findings provide new insights into sulfur metabolic diversity in hypersaline environments, underscoring the ecological role of sulfur-transforming microbes in sustaining biogeochemical processes under extreme conditions.

### 3.7 Mechanisms of osmoadaptation

**Figure 8:**
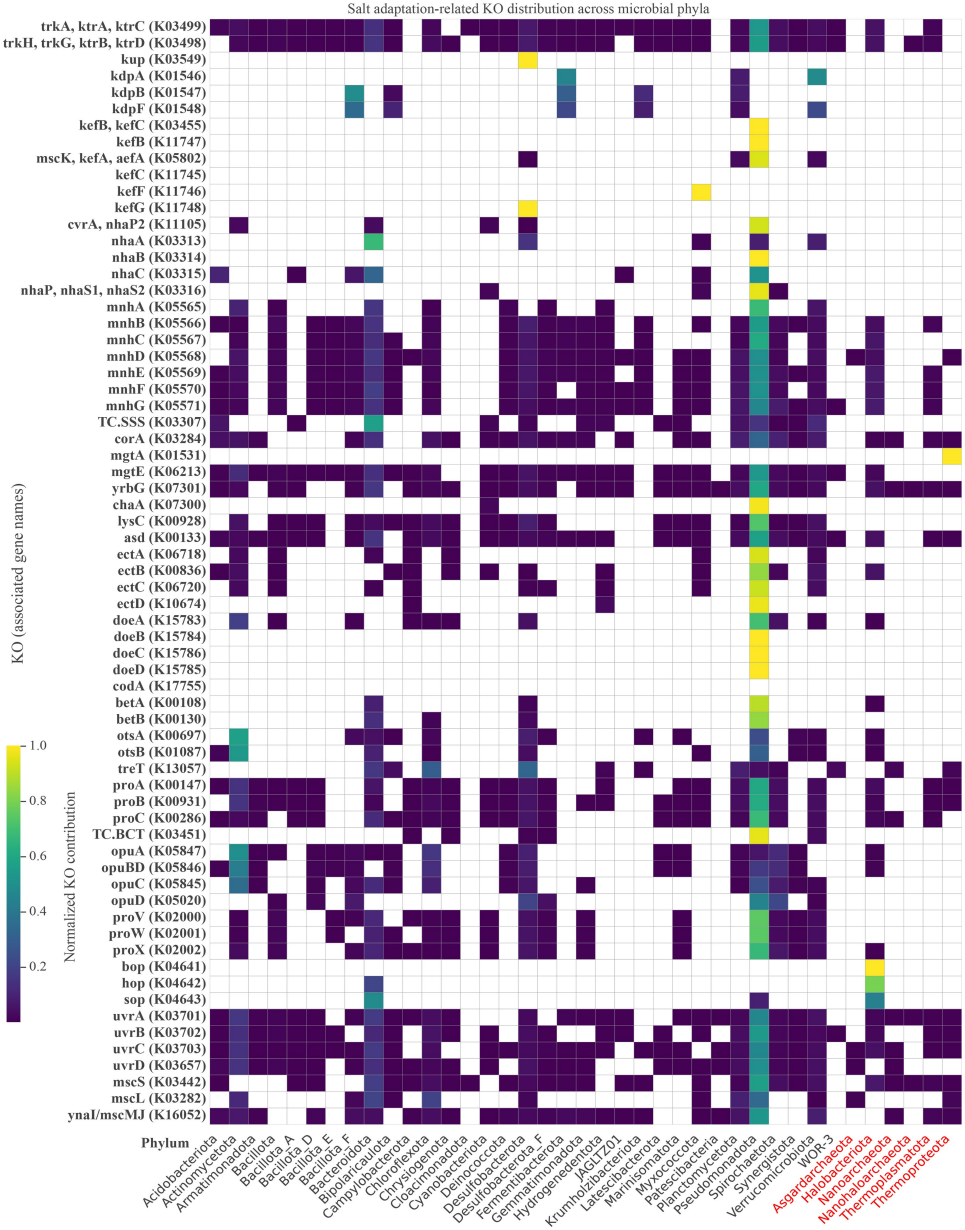
Heatmap showing the distribution of salt adaptation-related KEGG orthologs (KOs) across microbial phyla in Lake Barkol. KO abundance values are normalized and color-coded, with yellow indicating high relative contribution. Genes are grouped by function, including ion transporters, compatible solute synthesis and transport, mechanosensitive channels, rhodopsins, and stress response systems. Archaeal phyla are highlighted in red.

Microorganisms inhabiting hypersaline environments employ multiple strategies to maintain osmotic equilibrium under high salinity and fluctuating salt conditions. These strategies can be categorized into two main approaches, the salt-in strategy (salt sequestration) and the compatible solute strategy (salt-exclusion). In the salt-in strategy, microorganisms achieve osmotic equilibrium by accumulating high concentrations of inorganic salts or ions within their cytoplasm (Jensen et al., 2015; Gunde-Cimerman et al., 2018). Potassium (K^+^) is typically the primary cation, and chloride (Cl^−^) is the predominant anion stored intracellularly, while sodium (Na^+^) ions are generally expelled from the cells. High cytosolic potassium ions are less damaging to cellular enzymes compared to elevated levels of sodium ions (van de Vossenberg et al., 2000). This ion regulation is facilitated by mechanisms such as ATP-driven pumps, antiporters, or other transport systems that help manage ion transport across cellular membranes (Daoud and Ali, 2020). Among the salt adaptation-related functions, potassium uptake systems and Na^+^/H^+^ antiporters displayed approximately equal relative abundance across lake water and sediment metagenomes. This function represents a core mechanism for maintaining intracellular ionic balance under osmotic stress in Lake Barkol (Figure 8). Maintaining high intracellular potassium (K^+^) levels is essential for microbial survival. A number of studies have demonstrated that the Trk and Ktr uptake systems primarily respond to osmotic stress induced by elevated salinity and constitutes a pivotal component of salt stress in environments with higher salinity (Wu et al., 2024; Zhang et al., 2024). While Na^+^/H^+^ antiporters play a key role in ion homeostasis by expelling excess sodium (Na^+^) and regulating intracellular pH through proton exchange (Wang et al., 2023). The balanced representation of nhaA/B/C and mnh complex genes across both sample types indicates a shared requirement for sodium extrusion mechanisms in both planktonic and benthic microbial communities, despite differences in bulk salinity and sediment chemistry in Lake Barkol.

In this study, functional enrichment analysis, revealed that lake water microbiomes were significantly enriched in genes associated with compatible solute metabolism and photoactive energy generation. Specifically, genes encoding bacterial rhodopsin, sensory rhodopsin, and halorhodopsin were highly represented. These light-driven proton pumps may provide an auxiliary energy source under high osmotic stress. They help maintain ion homeostasis and cellular activity when conventional metabolic pathways are limited. Halophilic archaea rhodopsins function as key ion pumps that contribute to both energy production and pH regulation under extreme salinity conditions (Fotiadis et al., 2006). In Lake Barkol, *Halobacteriota*, *Pseudomonadota* and *Bacteroidota* were particularly enriched in genes encoding light-driven proton pumps, emphasizing the utilization of light-driven proton pumps is an auxiliary energy-conservation mechanism in the photic and osmotically dynamic water column.

The glycine betaine biosynthesis genes betA and betB were detected at relatively low abundance and were limited to a few phyla, including *Bacteroidota*, *Desulfobacterota*, *Pseudomonadota*, and *Halobacteriota*, genes encoding the ectoine biosynthesis pathway (ectA, ectB, ectC) exhibited broader phylogenetic representation, and were identified in MAGs affiliated with *Actinomycetota*, *Bacillota*, *Bacteroidota*, *Campylobacterota*, *Hydrogenedentota*, *Myxococcota*, *Pseudomonadota*, and *Verrucomicrobiota*. The final product, ectoine, may be further hydroxylated into 5-hydroxyectoine by ectoine hydroxylase (ectD), detected across multiple MAGs, particularly within the phyla *Campylobacterota*, *Hydrogenedentota*, and *Pseudomonadota*. Previous studies have demonstrated that *Pseudomonadota*, including *Pseudomonas stutzeri*, utilize ectD to produce hydroxyectoine as a dominant osmoprotectant (Stöveken et al., 2011). Similarly, halophilic *Gammaproteobacteria* like *Chromohalobacter salexigens* upregulate hydroxyectoine synthesis under heat and salt stress, underlining its dual role in osmoprotectant and thermoprotection (García-Estepa et al., 2006). The detection of ectD in *Hydrogenedentota* and *Campylobacterota* further expands the known distribution of hydroxyectoine biosynthesis capacity among extremophiles. Proline synthesis, and glycine betaine synthesis and transport (betA, betB, proVWX, opu operons) were notably enriched in lake water microbiomes, members of *Halobacteriota* possess diverse strategies for coping with osmotic stress, notably through *de novo* synthesis of trehalose and proline, as well as the uptake of compatible solutes such as glycine-betaine. Trehalose biosynthesis via the OtsAB pathway, has been shown to accumulate in low-salinity environments, offering membrane stabilization and protein protection. At higher salinities, cells preferentially uptake glycine-betaine, indicating a dynamic switch in osmoprotective strategies. The ectoine degradation genes (doeBDAC) were generally low in abundance, and several were absent from many dominant microbial phyla. Notably, *Pseudomonadota* was among the most abundant phyla. The widespread presence of trehalose and proline metabolism genes across *Halobacteriales* genomes suggests the critical role of organic osmolyte-mediated osmoprotection under hypersaline stress (Youssef et al., 2013). These groups are well known for their adaptability to hypersaline environments and likely rely on hydroxyectoine biosynthesis for maintaining cellular homeostasis under osmotic pressure. Similarly, proline and glycine betaine are well-established compatible solutes in halophiles, functioning as osmotically neutral intracellular solutes that help maintain turgor pressure and prevent dehydration (da Costa et al., 1998; Bremer and Krämer, 2000). These results suggest that halophilic archaea exhibit metabolic versatility, allowing for survival across a broad range of salinity levels and environmental fluctuations. The flexibility of microbial communities in switching between de novo synthesis and environmental uptake of such solutes to cope with fluctuating osmotic gradients. In addition to the osmoadaptive strategies described above, microorganisms inhabiting hypersaline environments must also cope with osmotic downshocks, such as sudden decreases in external salinity. Under such conditions, the cells are subjected to hypoosmotic stress, requiring rapid expulsion of accumulated compatible solutes to prevent cell lysis. One key mechanism for this response involves mechanosensitive (MS) channels, which act as emergency release valves to dissipate internal pressure by allowing ions and small solutes to exit the cell (Blount and Moe, 1999; Ramsey et al., 2024).

Among the MS channels, the small-conductance mechanosensitive channel (MscS, mscMJ), large-conductance mechanosensitive channel (MscL) in our metagenomic dataset, were present in a broader range of microbial phyla. Moreover, the presence of genes associated with stress-induced DNA repair systems indicates an additional layer of protection, enabling the repair of macromolecular damage triggered by osmotic and oxidative stress (Storz and Hengge-Aronis, 2000). These integrated responses reflect a multifaceted survival strategy in which microorganisms coordinate osmolyte metabolism, membrane transport, and genetic maintenance to sustain cellular viability under extreme environmental pressures. Collectively, these findings reveal a composite adaptation strategy whereby microorganisms integrate compatible solute metabolism, light-driven energy conservation, and ion homeostasis to maintain viability under extreme osmotic conditions in lake Barkol.

## 4 Conclusion

This study unravels the intricate interplay between environmental gradients and microbial functional partitioning in Lake Barkol, a hypersaline ecosystem characterized by pronounced redox and salinity stratification. Through metagenomic reconstruction of 309 MAGs, we revealed a taxonomically diverse microbial community dominated by novel, unclassified lineages (27 classes, 84 orders, 138 families, and 210 genera), underscoring the vast microbial “dark matter” thriving in this extreme habitat. These communities exhibited stark metabolic zonation: oxygenated surface waters hosted phototrophic carbon fixation via the CBB cycle, driven by *Cyanobacteriota* and chemolithoautotrophic *Gammaproteobacteria*, while anoxic sediments favored energy-efficient reductive pathways (WL and rTCA cycles) mediated by *Desulfobacterota*, *Chloroflexota*, and archaeal *Thermoplasmatota*. Such stratification highlights how redox and salinity gradients shape microbial resource partitioning, with methanogenic *Halobacteriota* further contributing to sedimentary carbon cycling under hypersaline constraints.

Compartmentalized nitrogen and sulfur cycling further emphasized habitat-specific adaptations. Nitrogen fixation and urea hydrolysis dominated the water column, whereas dissimilatory nitrate reduction (DNRA), denitrification, and ammonification prevailed in sediments, with *Pseudomonadota* serving as keystone taxa across these pathways. Sulfur metabolism mirrored this redox divide, with sulfur-oxidizing *Rhodobacteraceae* and sulfate-reducing *Desulfobacterota* mediating oxidative and reductive processes in water and sediments, respectively. These findings illustrate how microbial consortia partition biogeochemical roles to exploit niche-specific redox potentials, sustaining nutrient cycling in a hypersaline matrix.

Osmoadaptation strategies further delineated microbial niche specialization. Surface communities prioritized light-driven proton pumps (rhodopsins) and compatible solutes (ectoine, trehalose) for energy conservation and osmotic balance, whereas benthic lineages relied on ion transporters (Na^+^/H^+^ antiporters) and stress-resilient pathways (hydroxyectoine synthesis) to mitigate fluctuating salinity. The absence of canonical nitrification and methane oxidation pathways suggests hypersaline suppression of these processes, compensated by metabolic redundancy (DNRA) and cross-pathway interactions.

By integrating MAG-based functional annotation with environmental metadata, this study advances our understanding of microbial resilience in athalassohaline ecosystems. The discovery of unclassified lineages and niche-specific metabolic innovations highlights Lake Barkol as a reservoir of untapped microbial diversity and adaptive strategies. These insights not only refine models of biogeochemical cycling in extreme environments but also emphasize the need for multi-omics approaches (metatranscriptomics, proteomics) to resolve activity patterns and syntrophic networks. Future work should explore how fluctuating salinity and climate-driven perturbations modulate microbial interactions, with broader implications for ecosystem stability in global hypersaline lakes.

## Data Availability

The original contributions presented in the study are publicly available. This data can be found here: https://www.ncbi.nlm.nih.gov; BioProject: PRJNA1200918.

## Footnotes

^1^ https://www.bioinformatics.babraham.ac.uk/projects/fastqc/

^2^ https://github.com/wwood/CoverM

^3^ https://www.chiplot.online/

## References

[ref1] Alamoudi R. Barozzi A. Michoud G. van Goethem M. W. Odobel C. Chen Y.. (2025). Metabolic redundancy and specialisation of novel sulfide-oxidizing Sulfurimonas and Sulfurovum along the brine-seawater interface of the Kebrit deep. Environ. Microbiome 20:19. doi: 10.1186/s40793-025-00669-7, PMID: 39910644 PMC11800652

[ref2] Alneberg J. Bjarnason B. S. de Bruijn I. Schirmer M. Quick J. Ijaz U. Z.. (2014). Binning metagenomic contigs by coverage and composition. Nat. Methods 11, 1144–1146. doi: 10.1038/nmeth.3103, PMID: 25218180

[ref3] Andrews S. FastQC: A quality control tool for high throughput sequence data. (2010). Available online at: http://www.bioinformatics.babraham.ac.uk/projects/fastqc/.

[ref4] Aramaki T. Blanc-Mathieu R. Endo H. Ohkubo K. Kanehisa M. Goto S.. (2020). KofamKOALA: KEGG Ortholog assignment based on profile HMM and adaptive score threshold. Bioinformatics 36, 2251–2252. doi: 10.1093/bioinformatics/btz859, PMID: 31742321 PMC7141845

[ref5] Bar-Even A. Noor E. Milo R. (2012). A survey of carbon fixation pathways through a quantitative lens. J. Exp. Bot. 63, 2325–2342. doi: 10.1093/jxb/err417, PMID: 22200662

[ref6] Bay S. K. Dong X. Bradley J. A. Leung P. M. Grinter R. Jirapanjawat T.. (2021). Trace gas oxidizers are widespread and active members of soil microbial communities. Nat. Microbiol. 6, 246–256. doi: 10.1038/s41564-020-00811-w, PMID: 33398096

[ref7] Berg I. A. (2011). Ecological aspects of the distribution of different autotrophic CO2 fixation pathways. Appl. Environ. Microbiol. 77, 1925–1936. doi: 10.1128/AEM.02473-10, PMID: 21216907 PMC3067309

[ref8] Bernhard A. E. Donn T. Giblin A. E. Stahl D. A. (2005). Loss of diversity of ammonia-oxidizing bacteria correlates with increasing salinity in an estuary system. Environ. Microbiol. 7, 1289–1297. doi: 10.1111/j.1462-2920.2005.00808.x, PMID: 16104852

[ref9] Bertrand J. C. Caumette P. Lebaron P. Matheron R. Normand P. Sime-Ngando T. (2015). Biogeochemical cycles environmental microbiology: Fundamentals and applications. Dordrecht: Springer.

[ref9001] Bhatnagar S. Cowley E. S. Kopf S. H. Pérez Castro S. Kearney S. Dawson S. C.. (2020). Microbial community dynamics and coexistence in a sulfide-driven phototrophic bloom. Environ. Microbiol. 15, 1–17. doi: 10.1186/s40793-019-0348-0 PMC8066431 33902727

[ref10] Blount P. Moe P. C. (1999). Bacterial mechanosensitive channels: integrating physiology, structure and function. Trends Microbiol. 7, 420–424. doi: 10.1016/s0966-842x(99)01594-2, PMID: 10498951

[ref11] Boey J. S. Mortimer R. Couturier A. Worrallo K. Handley K. M. (2022). Estuarine microbial diversity and nitrogen cycling increase along sand-mud gradients independent of salinity and distance. Environ. Microbiol. 24, 50–65. doi: 10.1111/1462-2920.15550 33973326

[ref12] Bolger A. M. Lohse M. Usadel B. (2014). Trimmomatic: a flexible trimmer for Illumina sequence data. Bioinformatics 30, 2114–2120. doi: 10.1093/bioinformatics/btu170, PMID: 24695404 PMC4103590

[ref13] Braga R. M. Dourado M. N. Araújo W. L. (2016). Microbial interactions: ecology in a molecular perspective. Braz. J. Microbiol. 47, 86–98. doi: 10.1016/j.bjm.2016.10.005, PMID: 27825606 PMC5156507

[ref14] Bremer E. Krämer R. (2000). “Coping with osmotic challenges: osmoregulation through accumulation and release of compatible solutes in Bacteria” in Bacterial stress responses. eds. Storz G. Hengge-Aronis R. (Washington, D.C.: American Society for Microbiology Press), 79–97.

[ref15] Cai W. Yu K. Yang W. Mu R. Lian C. Xie L.. (2023). Prokaryotic community structure, abundances, and potential ecological functions in a Mars analog salt-lake. Astrobiology 23, 550–562. doi: 10.1089/ast.2022.0091, PMID: 37130293

[ref9002] Chaumeil P. A. Mussig A. J. Hugenholtz P. Parks D. H. (2020). GTDB-Tk: a toolkit to classify genomes with the Genome Taxonomy Database. Bioinform. doi: 10.1093/bioinformatics/btz848 PMC7703759 31730192

[ref16] Cordero P. R. Bayly K. Man Leung P. Huang C. Islam Z. F. Schittenhelm R. B.. (2019). Atmospheric carbon monoxide oxidation is a widespread mechanism supporting microbial survival. ISME J. 13, 2868–2881. doi: 10.1038/s41396-019-0479-8, PMID: 31358912 PMC6794299

[ref17] da Costa M. S. Santos H. Galinski E. A. (1998). An overview of the role and diversity of compatible solutes in Bacteria and Archaea. Adv. Biochem. Eng. Biotechnol. 61, 117–153. doi: 10.1007/BFb0102291, PMID: 9670799

[ref18] Daoud L. Ali M. B. (2020). Halophilic microorganisms: interesting group of extremophiles with important applications in biotechnology and environment. In Physiological and biotechnological aspects of extremophiles (Academic Press), 51–64. doi: 10.1016/B978-0-12-818322-9.00005-8

[ref19] Ebenhöh O. Spelberg S. (2018). The importance of the photosynthetic Gibbs effect in the elucidation of the Calvin–Benson–Bassham cycle. Biochem. Soc. Trans. 46, 131–140. doi: 10.1042/BST20170245, PMID: 29305411 PMC5818666

[ref20] Fang Y. Liu J. Yang J. Wu G. Hua Z. Dong H.. (2022). Compositional and metabolic responses of autotrophic microbial community to salinity in lacustrine environments. mSystems 7:e0033522. doi: 10.1128/msystems.00335-22, PMID: 35862818 PMC9426519

[ref21] Gao L. Fang B. Z. Liu Y. H. Jiao J. Y. Li M. M. Antunes A.. (2022). *Rhabdothermincola salaria* sp. nov., a novel actinobacterium isolated from a saline lake sediment. Int. J. Syst. Evol. Microbiol. 72:005361. doi: 10.1099/ijsem.0.005361 35544312

[ref22] Gao L. She T. T. Liu Y. H. Chen Z. Y. Liu J. Y. Jiang H. C.. (2024). *Chelativorans salis* sp. nov., a slightly halophilic bacterium isolated from an enrichment system with saline lake sediment. Int. J. Syst. Evol. Microbiol. 74:006340. doi: 10.1099/ijsem.0.006340 38619977

[ref23] García-Estepa R. Argandoña M. Reina-Bueno M. Capote N. Iglesias-Guerra F. Nieto J. J.. (2006). The ectD gene, which is involved in the synthesis of the compatible solute hydroxyectoine, is essential for thermoprotection of the halophilic bacterium *Chromohalobacter salexigens*. J. Bacteriol. 188, 3774–3784. doi: 10.1128/JB.00136-06, PMID: 16707670 PMC1482885

[ref24] Garrity G. M. Stanley J. T. (2001) in Bergey’s manual® of systematic bacteriology: Volume one the Archaea and the deeply branching and phototrophic Bacteria. eds. Boone D. R. Castenholz R. W. (New York: Springer).

[ref25] Giri B. J. Bano N. Hollibaugh J. T. (2004). Distribution of RuBisCO genotypes along a redox gradient in mono Lake, California. Appl. Environ. Microbiol. 70, 3443–3448. doi: 10.1128/AEM.70.6.3443-3448.2004, PMID: 15184142 PMC427752

[ref26] Gorrasi S. Pasqualetti M. Braconcini M. Muñoz-Palazon B. Fenice M. (2022). Could Pontimonas harbour halophilic members able to withstand very broad salinity variations. Microorganisms 10:790. doi: 10.3390/microorganisms10040790, PMID: 35456840 PMC9030170

[ref7001] Gruber N. (2008). Nitrogen in the Marine Environment, (eds.) Capone D. G. Bronk D. Mulholland M. Carpenter E. J., (Academic Press), ed. 2, 1–15.

[ref27] Gruber N. Galloway J. N. (2008). An earth-system perspective of the global nitrogen cycle. Nature 451, 293–296. doi: 10.1038/nature06592, PMID: 18202647

[ref28] Gunde-Cimerman N. Plemenitaš A. Oren A. (2018). Strategies of adaptation of microorganisms of the three domains of life to high salt concentrations. FEMS Microbiol. Rev. 42, 353–375. doi: 10.1093/femsre/fuy009, PMID: 29529204

[ref29] Gurevich A. Saveliev V. Vyahhi N. Tesler G. (2013). QUAST: quality assessment tool for genome assemblies. Bioinformatics 29, 1072–1075. doi: 10.1093/bioinformatics/btt086, PMID: 23422339 PMC3624806

[ref9003] Han S. B. Yu Y. H. Ju Z. Li Y. Zhang R. Hou X. J.. (2018). Rhodohalobacter barkolensis sp. nov., isolated from a saline lake and emended description of the genus Rhodohalobacter. Int. J. Syst. Evol. Microbiol. 68, 1949–1954. doi: 10.1099/ijsem.0.002775 29676726

[ref30] Handelsman J. Rondon M. R. Brady S. F. Clardy J. Goodman R. M. (1998). Molecular biological access to the chemistry of unknown soil microbes: a new frontier for natural products. Chem. Biol. 5, R245–R249. doi: 10.1016/s1074-5521(98)90108-9, PMID: 9818143

[ref9004] Hyatt D. Chen G. L. LoCascio P. F. Land M. L. Larimer F. W. Hauser L. J. (2010). Prodigal: prokaryotic gene recognition and translation initiation site identification. BMC Bioinform. 11:119. doi: 10.1186/1471-2105-11-119 PMC2848648 20211023

[ref31] Fotiadis D. Jastrzebska B. Philippsen A. Müller D. J. Palczewski K. Engel A. (2006). Structure of the rhodopsin dimer: a working model for G-protein-coupled receptors. Curr. Opin. Struct. Biol. 16, 252–259. doi: 10.1016/j.sbi.2006.03.013 16567090

[ref32] Holmer M. Storkholm P. (2001). Sulphate reduction and Sulphur cycling in lake sediments: a review. Freshw. Biol. 46, 431–451. doi: 10.1046/j.1365-2427.2001.00687.x

[ref33] Hügler M. Sievert S. M. (2011). Beyond the Calvin cycle: autotrophic carbon fixation in the ocean. Annu. Rev. Mar. Sci. 3, 261–289. doi: 10.1146/annurev-marine-120709-142712, PMID: 21329206

[ref34] Jensen M. W. Matlock S. A. Reinheimer C. H. Lawlor C. J. Reinheimer T. A. Gorrell A. (2015). Potassium stress growth characteristics and energetics in the haloarchaeon *Haloarcula marismortui*. Extremophiles 19, 315–325. doi: 10.1007/s00792-014-0716-z, PMID: 25503059 PMC4339784

[ref9005] Jia T. Niu L. Qi Z. Cong W. Xi J. Yang C.. (2025). The biosynthesis, storage and utilization of elemental sulfur: Enzymatic pathways, molecular mechanisms, and future perspectives. Crit. Rev. Environ. Sci. Technol. 55, 483–506. doi: 10.1080/10643389.2024.2421087

[ref35] Johnson S. S. Chevrette M. G. Ehlmann B. L. Benison K. C. (2015). Insights from the metagenome of an acid salt-lake: the role of biology in an extreme depositional environment. PLoS One 10:e0122869. doi: 10.1371/journal.pone.0122869, PMID: 25923206 PMC4414474

[ref36] Kang D. D. Li F. Kirton E. Thomas A. Egan R. An H.. (2019). MetaBAT 2: an adaptive binning algorithm for robust and efficient genome reconstruction from metagenome assemblies. PeerJ 7:e7359. doi: 10.7717/peerj.7359, PMID: 31388474 PMC6662567

[ref9006] Kanehisa M. Goto S. (2000). KEGG: kyoto encyclopedia of genes and genomes. Nucleic acids research, 28, 27–30. doi: 10.1093/nar/28.1.27 10592173 PMC102409

[ref9007] King G. M. Weber C. F. (2007). Distribution, diversity and ecology of aerobic CO-oxidizing bacteria. Nat. Rev. Microbiol. 5, 107–118. doi: 10.1038/nrmicro1595 17224920

[ref37] Kovaleva O. L. Tourova T. P. Muyzer G. Kolganova T. V. Sorokin D. Y. (2011). Diversity of RuBisCO and ATP citrate lyase genes in soda lake sediments. FEMS Microbiol. Ecol. 75, 37–47. doi: 10.1111/j.1574-6941.2010.00996.x, PMID: 21073490

[ref38] La Cono V. Ninh K. B. Yakimov M. M. (2024). Unique features of extremely halophilic microbiota inhabiting solar saltwork fields of Vietnam. Preprints. doi: 10.20944/preprints202408.2086.v1 PMC11509607 39458284

[ref39] Leung P. M. Grinter R. Tudor-Matthew E. Lingford J. P. Jimenez L. Lee H. C.. (2024). Trace gas oxidation sustains energy needs of a thermophilic archaeon at suboptimal temperatures. Nat. Commun. 15:3219. doi: 10.1038/s41467-024-47324-2, PMID: 38622143 PMC11018855

[ref40] Li Y. Q. Chai Y. H. Wang X. S. Huang L. Y. Luo X. M. Qiu C.. (2021). Bacterial community in saline farmland soil on the Tibetan plateau: responding to salinization while resisting extreme environments. BMC Microbiol. 21:119. doi: 10.1186/s12866-021-02190-6, PMID: 33874905 PMC8056723

[ref9008] Li H. Durbin R.. (2009). “Fast and accurate short read alignment with burrows-wheeler transform.” Bioinform. 25, 1754–1760. doi: 10.1093/bioinformatics/btp324 PMC2705234 19451168

[ref7002] Li W. Godzik A. (2006). Cd-hit: a fast program for clustering and comparing large sets of protein or nucleotide sequences. Bioinformatics (Oxford, England), 22, 1658–1659. doi: 10.1093/bioinformatics/btl158 16731699

[ref9009] Li H. Handsaker B. Wysoker A. Fennell T. Ruan J. Homer N. (2009). The sequence alignment/map format and SAMtools. Bioinform. 25, 2078–2079. doi: 10.1093/bioinformatics/btp352 PMC2723002 19505943

[ref41] Li D. Liu C. M. Luo R. Sadakane K. Lam T. W. (2015). MEGAHIT: an ultra-fast single-node solution for large and complex metagenomics assembly via succinct de Bruijn graph. Bioinformatics 31, 1674–1676. doi: 10.1093/bioinformatics/btv033 25609793

[ref7003] Li D. Luo R. Liu C. M. Leung C. M. Ting H. F. Sadakane K.. (2016). MEGAHIT v1.0: A fast and scalable metagenome assembler driven by advanced methodologies and community practices. Methods (San Diego, Calif.), 102, 3–11. doi: 10.1016/j.ymeth.2016.02.020 27012178

[ref42] Li L. Yue F. Li Y. Yang A. Li J. Lv Y.. (2020). Degradation pathway and microbial mechanism of high-concentration thiocyanate in gold mine tailings wastewater. RSC Adv. 10, 25679–25684. doi: 10.1039/D0RA03330H, PMID: 35518587 PMC9055349

[ref43] Liang H. Wang F. Mu R. Huang J. Zhao R. Li X.. (2021). Metagenomics analysis revealing the occurrence of antibiotic resistome in salt lakes. Sci. Total Environ. 790:148262. doi: 10.1016/j.scitotenv.2021.148262, PMID: 34380279

[ref44] Litchman E. Edwards K. F. Klausmeier C. A. (2015). Microbial resource utilization traits and trade-offs: implications for community structure, functioning, and biogeochemical impacts at present and in the future. Front. Microbiol. 6:254. doi: 10.3389/fmicb.2015.00254, PMID: 25904900 PMC4389539

[ref9010] Liu Y. H. Gao L. Jiang H. C. Fang B. Z. Huang Y. Li L.. (2024). Response of microbial diversity and function to the degradation of Barkol Saline Lake. Front. Microbiol. 15:1358222. doi: 10.3389/fmicb.2024.1358222 38784797 PMC11111964

[ref45] Liu W. Jiang H. Yang J. Wu G. (2018). Gammaproteobacterial diversity and carbon utilization in response to salinity in the lakes on the Qinghai-Tibetan plateau. Geomicrobiol J. 35, 392–403. doi: 10.1080/01490451.2017.1378951

[ref46] Liu Y. H. Mohamad O. A. A. Gao L. Xie Y. G. Abdugheni R. Huang Y.. (2023). Sediment prokaryotic microbial community and potential biogeochemical cycle from saline lakes shaped by habitat. Microbiol. Res. 270:127342. doi: 10.1016/j.micres.2023.127342, PMID: 36848700

[ref47] Liu Y. X. Qin Y. Chen T. Lu M. Qian X. Guo X.. (2021). A practical guide to amplicon and metagenomic analysis of microbiome data. Protein Cell 12, 315–330. doi: 10.1007/s13238-020-00724-8, PMID: 32394199 PMC8106563

[ref48] Lu Y. An C. Zhang J. Wang Z. Tao S. Zhao J. (2012). A Holocene dust record in arid Central Asia inferred from Lake Barkol, Northwest China. Environ. Earth Sci. 65, 213–220. doi: 10.1007/s12665-011-1084-y

[ref49] Ma Z. Wang Z. Liu J. Yuan B. Xiao J. Zhang G. (2004). U-series chronology of sediments associated with late quaternary fluctuations, Balikun Lake, northwestern China. Quat. Int. 121, 89–98. doi: 10.1016/j.quaint.2004.01.025

[ref50] Mackenzie F. T. Vink S. Wollast R. Chou L. (1995). “Comparative geochemistry of marine saline lakes” in Physics and chemistry of lakes. eds. Lerman A. Imboden D. M. Gat J. R. (Berlin, Heidelberg: Springer), 277–300.

[ref9011] Matsen F. A. Kodner R. B. Armbrust E. V. (2010). Pplacer: linear time maximum-likelihood and Bayesian phylogenetic placement of sequences onto a fixed reference tree. BMC Bioinform. 11:538. doi: 10.1186/1471-2105-11-538 PMC3098090 21034504

[ref51] McGonigle J. M. Bernau J. A. Bowen B. B. Brazelton W. J. (2022). Metabolic potential of microbial communities in the hypersaline sediments of the Bonneville salt flats. mSystems 7:e0084622. doi: 10.1128/msystems.00846-22, PMID: 36377900 PMC9765009

[ref52] Mobberley J. M. Khodadad C. L. Foster J. S. (2013). Metabolic potential of lithifying cyanobacteria-dominated thrombolitic mats. Photosynth. Res. 118, 125–140. doi: 10.1007/s11120-013-9890-6, PMID: 23868401 PMC5766932

[ref53] Muyzer G. Stams A. J. (2008). The ecology and biotechnology of sulphate-reducing bacteria. Nat. Rev. Microbiol. 6, 441–454. doi: 10.1038/nrmicro1892, PMID: 18461075

[ref9012] Nayfach S. Pollard K. S. (2016). Toward accurate and quantitative comparative metagenomics. Cell. 166, 1103–1116. doi: 10.1016/j.cell.2016.08.007 27565341 PMC5080976

[ref54] Olm M. R. Brown C. T. Brooks B. Banfield J. F. (2017). dRep: a tool for fast and accurate genomic comparisons that enables improved genome recovery from metagenomes through de-replication. ISME J. 11, 2864–2868. doi: 10.1038/ismej.2017.126, PMID: 28742071 PMC5702732

[ref55] Oren A. (2011). Thermodynamic limits to microbial life at high salt concentrations. Environ. Microbiol. 13, 1908–1923. doi: 10.1111/j.1462-2920.2010.02365.x, PMID: 21054738

[ref9013] Parks D. H. Imelfort M. Skennerton C. T. Hugenholtz P. Tyson G. W. (2015). CheckM: assessing the quality of microbial genomes recovered from isolates, single cells, and metagenomes. Genome Res. 25, 1043–1055. doi: 10.1101/gr.186072.114 25977477 PMC4484387

[ref56] Pavloudi C. Zafeiropoulos H. (2022). Deciphering the community structure and the functional potential of a hypersaline marsh microbial mat community. FEMS Microbiol. Ecol. 98:fiac141. doi: 10.1093/femsec/fiac141, PMID: 36416806

[ref57] Ponomarova O. Patil K. R. (2015). Metabolic interactions in microbial communities: untangling the Gordian knot. Curr. Opin. Microbiol. 27, 37–44. doi: 10.1016/j.mib.2015.06.014, PMID: 26207681

[ref58] Qiu Z. Zhu Y. Zhang Q. Qiao X. Mu R. Xu Z.. (2024). Unravelling biosynthesis and biodegradation potentials of microbial dark matters in hypersaline lakes. Environ. Sci. Ecotechnol. 20:100359. doi: 10.1016/j.ese.2023.100359, PMID: 39221074 PMC11361885

[ref59] Ramsey K. Britt M. Maramba J. Ushijima B. Moller E. Anishkin A.. (2024). The dynamic hypoosmotic response of *Vibrio cholerae* relies on the mechanosensitive channel MscS. iScience:27(6). doi: 10.1016/j.isci.2024.107364 PMC11167432 38868203

[ref60] Riesenfeld C. S. Schloss P. D. Handelsman J. (2004). Metagenomics: genomic analysis of microbial communities. Annu. Rev. Genet. 38, 525–552. doi: 10.1146/annurev.genet.38.072902.091216, PMID: 15568985

[ref61] Saccò M. White N. E. Harrod C. Salazar G. Aguilar P. Cubillos C. F.. (2021). Salt to conserve: a review on the ecology and preservation of hypersaline ecosystems. Biol. Rev. 96, 2828–2850. doi: 10.1111/brv.12780 34747117

[ref9014] Seemann T. (2014). Prokka: rapid prokaryotic genome annotation. Bioinform, 30, 2068–2069. doi: 10.1093/bioinformatics/btu153 24642063

[ref62] Sierra M. A. Ryon K. A. Tierney B. T. Foox J. Bhattacharya C. Afshin E.. (2022). Microbiome and metagenomic analysis of Lake hillier Australia reveals pigment-rich polyextremophiles and wide-ranging metabolic adaptations. Environ. Microbiome 17:60. doi: 10.1186/s40793-022-00455-9, PMID: 36544228 PMC9768965

[ref63] Sorokin D. Y. Tourova T. P. Lysenko A. M. Muyzer G. (2006). Diversity of culturable halophilic sulfur-oxidizing bacteria in hypersaline habitats. Microbiology 152, 3013–3023. doi: 10.1099/mic.0.29106-0, PMID: 17005982

[ref64] Storz G. Hengge-Aronis R. (2000). Bacterial stress responses. Washington, D.C.: ASM Press.

[ref65] Stöveken N. Pittelkow M. Sinner T. Jensen R. A. Heider J. Bremer E. (2011). A specialized aspartokinase enhances the biosynthesis of the osmoprotectants ectoine and hydroxyectoine in *Pseudomonas stutzeri* A1501. J. Bacteriol. 193, 4456–4468. doi: 10.1128/JB.00345-11, PMID: 21725014 PMC3165526

[ref66] Uritskiy G. V. DiRuggiero J. Taylor J. (2018). MetaWRAP: a flexible pipeline for genome-resolved metagenomic data analysis. Microbiome 6:158. doi: 10.1186/s40168-018-0541-1, PMID: 30219103 PMC6138922

[ref67] van de Vossenberg J. L. Driessen A. J. Konings W. N. (2000). “Adaptations of the cell membrane for life in extreme environments cell and molecular response to stress” in Cell and Molecular Response to Stress, vol. 1 (Elsevier), 71–88.

[ref68] Vavourakis C. D. Andrei A. S. Mehrshad M. Ghai R. Sorokin D. Y. Muyzer G. (2018). A metagenomics roadmap to the uncultured genome diversity in hypersaline soda lake sediments. Microbiome 6, 1–18. doi: 10.1186/s40168-018-0548-7, PMID: 30231921 PMC6146748

[ref69] Vavourakis C. D. Ghai R. Rodriguez-Valera F. Sorokin D. Y. Tringe S. G. Hugenholtz P.. (2016). Metagenomic insights into the uncultured diversity and physiology of microbes in four hypersaline soda lake brines. Front. Microbiol. 7:211. doi: 10.3389/fmicb.2016.00211, PMID: 26941731 PMC4766312

[ref70] Wang L. Lian C. Wan W. Qiu Z. Luo X. Huang Q.. (2023). Salinity-triggered homogeneous selection constrains the microbial function and stability in lakes. Appl. Microbiol. Biotechnol. 107, 6591–6605. doi: 10.1007/s00253-023-12696-w, PMID: 37688597

[ref71] Wood A. P. Kelly D. P. (1991). Isolation and characterisation of *Thiobacillus halophilus* sp. nov., a Sulphur-oxidising autotrophic eubacterium from a Western Australian hypersaline lake. Arch. Microbiol. 156, 277–280. doi: 10.1007/BF00262998

[ref72] Wu Z. Li M. Qu L. Zhang C. Xie W. (2024). Metagenomic insights into microbial adaptation to the salinity gradient of a typical short residence-time estuary. Microbiome 12:115. doi: 10.1186/s40168-024-01817-w, PMID: 38918820 PMC11200988

[ref73] Wu Y. W. Simmons B. A. Singer S. W. (2016). MaxBin 2.0: an automated binning algorithm to recover genomes from multiple metagenomic datasets. Bioinformatics 32, 605–607. doi: 10.1093/bioinformatics/btv638, PMID: 26515820

[ref74] Youssef N. Savage-Ashlock K. McCully A. Luedtke B. Shaw E. I. Hoff W. D.. (2013). Trehalose/2-sulfotrehalose biosynthesis and glycine-betaine uptake are widely spread mechanisms for osmoadaptation in the Halobacteriales. ISME J. 8, 636–649. doi: 10.1038/ismej.2013.165, PMID: 24048226 PMC3930309

[ref9015] Zhang M. Xing J. Long Q. Shen G. Zhu D. Li Y. (2024). Prokaryotic microbial diversity analysis and preliminary prediction of metabolic function in salt lakes on the Qinghai–Tibet Plateau. Water. 16:451. doi: 10.3390/w16030451

[ref75] Zheng X. (1987). Salt lakes and their origins in Xinjiang, China. Chin. J. Oceanol. Limnol. 5, 172–185. doi: 10.1007/BF02845001, PMID: 40196704

[ref76] Zhou Z. Tran P. Q. Breister A. M. Liu Y. Kieft K. Cowley E. S.. (2022). METABOLIC: high-throughput profiling of microbial genomes for functional traits, metabolism, biogeochemistry, and community-scale functional networks. Microbiome 10:33. doi: 10.1186/s40168-021-01213-8, PMID: 35172890 PMC8851854

[ref77] Zuñiga C. Zaramela L. Zengler K. (2017). Elucidation of complexity and prediction of interactions in microbial communities. Microb. Biotechnol. 10, 1500–1522. doi: 10.1111/1751-7915.12855, PMID: 28925555 PMC5658597

