# Supplementary material for: Metagenomics-assembled genomes reveal microbial metabolic adaptation to athalassohaline environment, the case Lake Barkol, China
Source: Front Microbiol. 2025 Jun 4;16:1550346. doi: 10.3389/fmicb.2025.1550346 (PMC12174138; doi:10.3389/fmicb.2025.1550346)
Supplement: Supplementary file 2 [file Data_Sheet_1.pdf]

Metagenome-Assembled Genomes Reveal Microbial Community Metabolic Potential and Salt Adaptation in Extreme Saline Environments

Maripat Xamxidin[1], Xuanqi Zhang[2], Gang Zheng[3], Can Chen[1*] and Min Wu[1*]

1 College of Life Sciences, Zhejiang University, Hangzhou, China

2 College of Architecture and Engineering, Zhejiang University, Hangzhou, China

3 Ocean Research Center of Zhoushan, Zhejiang University, Zhoushan, China.

Correspondence

Min Wu*

wumin@zju.edu.cn.

Can Chen

chen_can_1990@163.com

Figure S1. Heatmap showing the variation in abundance of functional genes involved in carbon cycling pathways across water (W_1, W_4, W_8) and sediment (S_1, S_2, S_3) samples from Lake Barkol. Genes cover carbon fixation (CBB, WL, rTCA), fermentation, methanogenesis, and phototrophy. Marker genes for carbon fixation pathways are indicated with asterisks (*). Statistical differences between groups were calculated using Welch's t-test; *P < 0.05, **P < 0.01. Gene abundance is shown as counts per million (CPM).

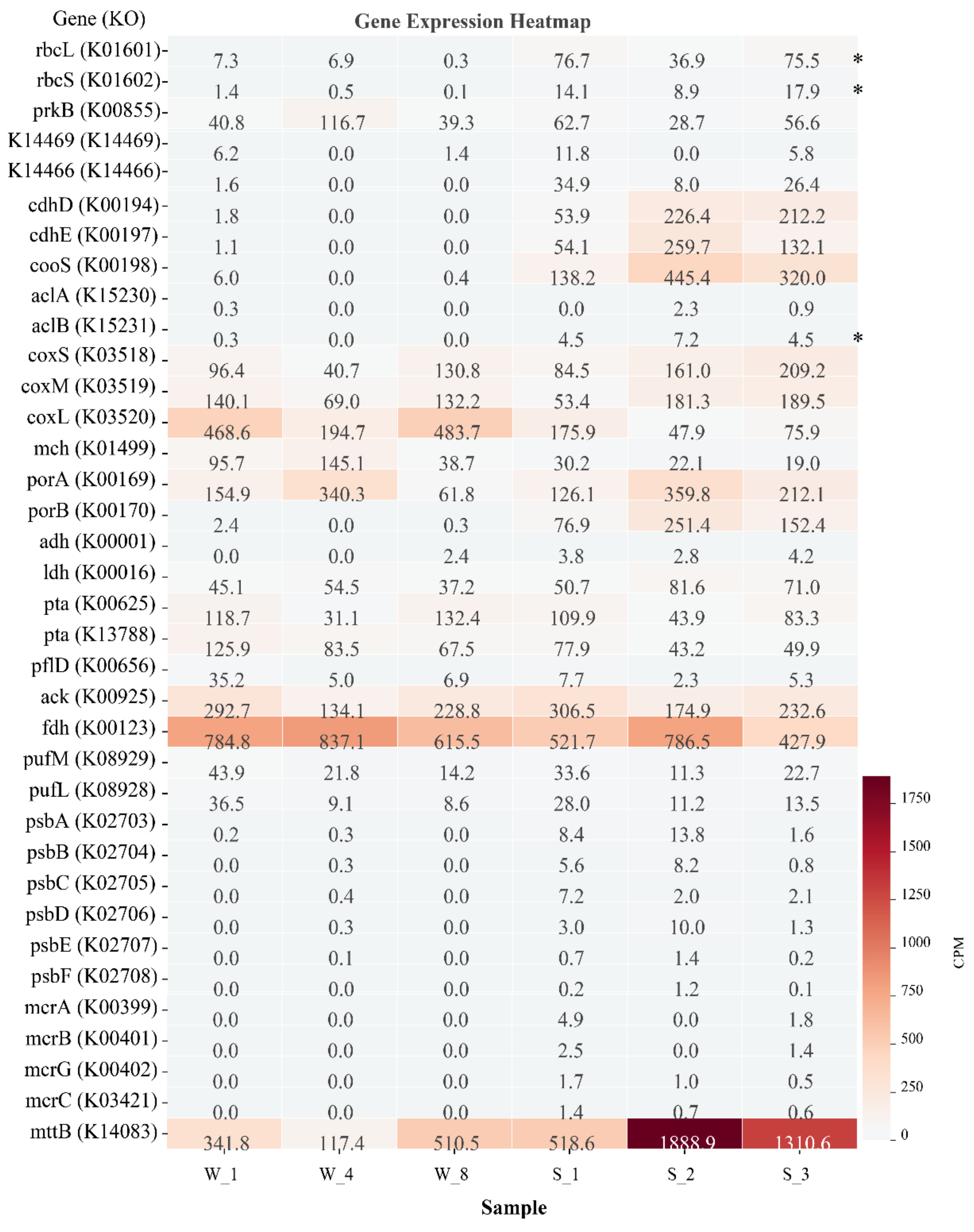

Figure S2. Heatmap showing the variation in abundance of functional genes involved in nitrogen metabolic pathways across water (W_1, W_4, W_8) and sediment (S_1, S_2, S_3) samples from Lake Barkol. Genes represent processes including nitrogen fixation, denitrification, dissimilatory nitrate reduction, and urea metabolism. Statistical significance between water and sediment groups was determined using Welch's t-test; *P < 0.05, **P < 0.01. Abundance values are represented as normalized gene counts (CPM).

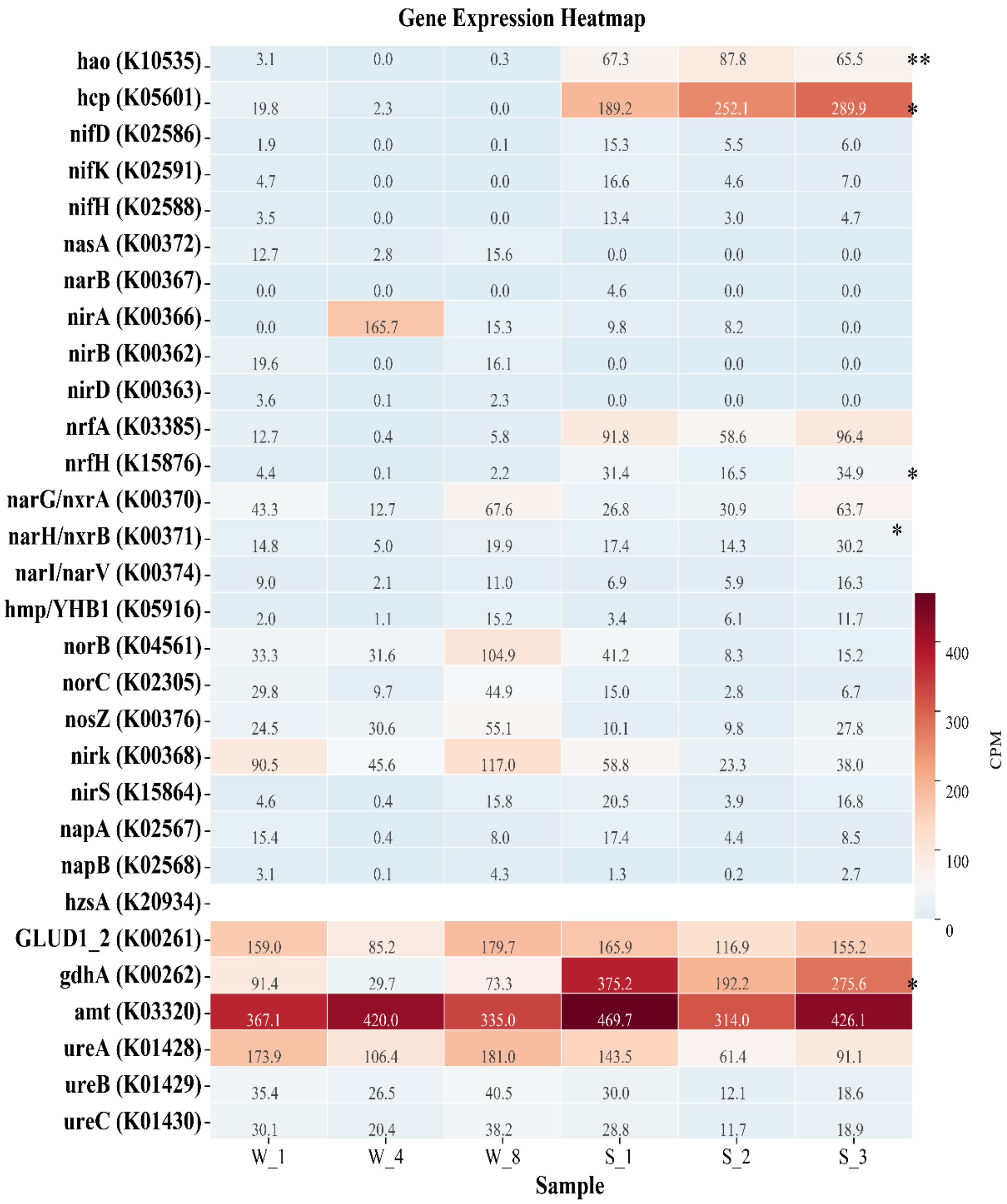

Figure S3. Heatmap showing the variation in abundance of functional genes involved in sulfur metabolic pathways across water (W_1, W_4, W_8) and sediment (S_1, S_2, S_3) samples from Lake Barkol. Functional categories include assimilatory sulfate reduction, dissimilatory sulfate reduction, sulfur/thiosulfate oxidation, and sulfite metabolism. Statistical significance between groups was assessed using Welch's t-test; *P < 0.05, **P < 0.01. Abundance values are represented as normalized gene counts (CPM).

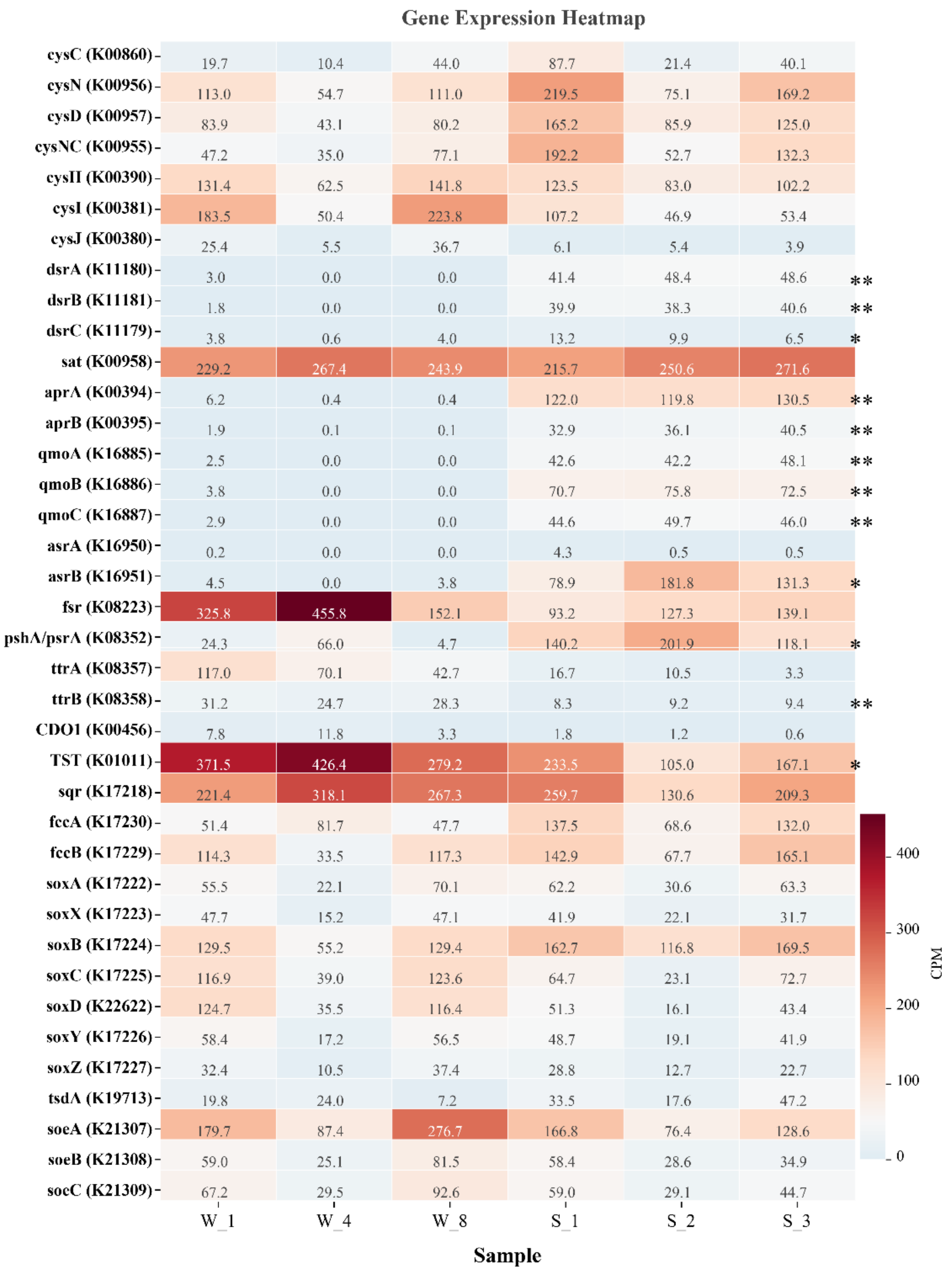

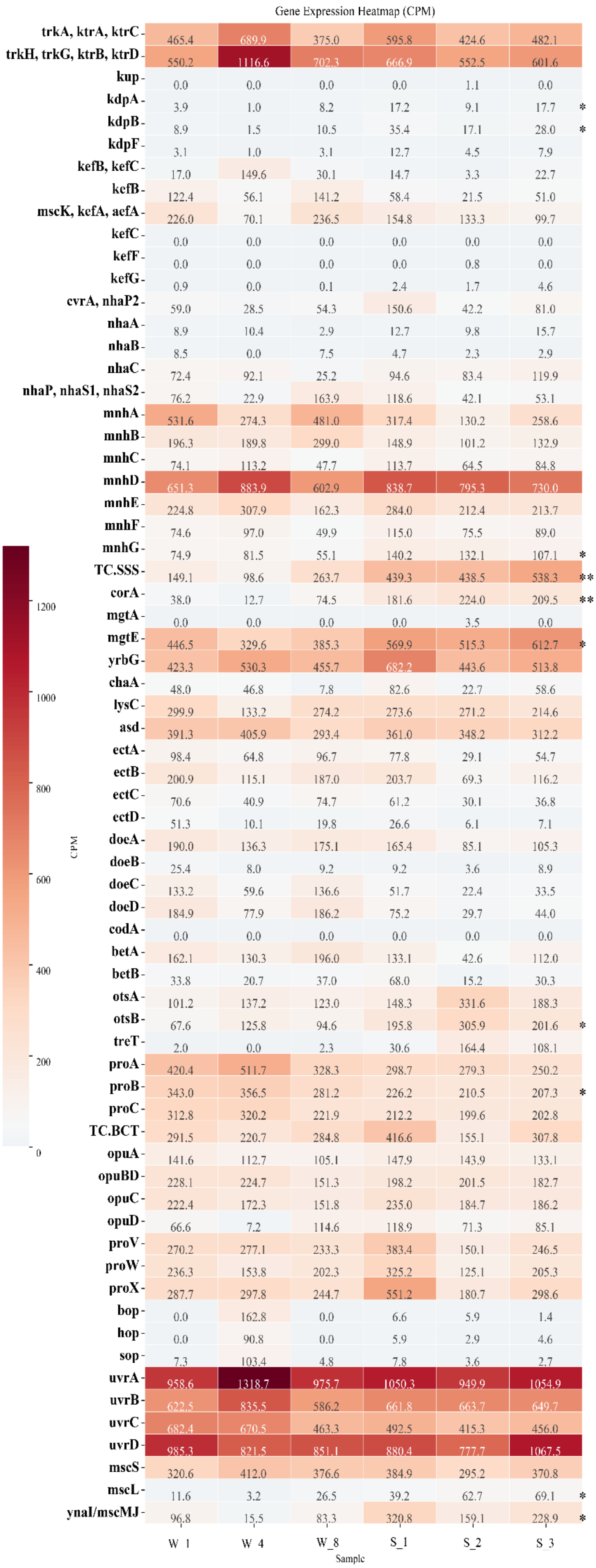
Gene Expression Heatmap (CPM)
W_1 W_4 W_8 S_1 S_2 S_3
Sample
CPM
0 200 400 600 800 1000 1200
trkA, ktrA, ktrC 465.4 689.9 375.0 595.8 424.6 482.1
trkH, trkG, ktrB, ktrD 550.2 1116.6 702.3 666.9 552.5 601.6
kup 0.0 0.0 0.0 0.0 1.1 0.0
kdpA 3.9 1.0 8.2 17.2 9.1 17.7 *
kdpB 8.9 1.5 10.5 35.4 17.1 28.0 *
kdpF 3.1 1.0 3.1 12.7 4.5 7.9
kefB, kefC 17.0 149.6 30.1 14.7 3.3 22.7
kefB 122.4 56.1 141.2 58.4 21.5 51.0
mscK, kefA, aefA 226.0 70.1 236.5 154.8 133.3 99.7
kefC 0.0 0.0 0.0 0.0 0.0 0.0
kefF 0.0 0.0 0.0 0.0 0.8 0.0
kefG 0.9 0.0 0.1 2.4 1.7 4.6
cvrA, nhaP2 59.0 28.5 54.3 150.6 42.2 81.0
nhaA 8.9 10.4 2.9 12.7 9.8 15.7
nhaB 8.5 0.0 7.5 4.7 2.3 2.9
nhaC 72.4 92.1 25.2 94.6 83.4 119.9
nhaP, nhaS1, nhaS2 76.2 22.9 163.9 118.6 42.1 53.1
mnhA 531.6 274.3 481.0 317.4 130.2 258.6
mnhB 196.3 189.8 299.0 148.9 101.2 132.9
mnhC 74.1 113.2 47.7 113.7 64.5 84.8
mnhD 651.3 883.9 602.9 838.7 795.3 730.0
mnhE 224.8 307.9 162.3 284.0 212.4 213.7
mnhF 74.6 97.0 49.9 115.0 75.5 89.0
mnhG 74.9 81.5 55.1 140.2 132.1 107.1 *
TC.SSS 149.1 98.6 263.7 439.3 438.5 538.3 **
corA 38.0 12.7 74.5 181.6 224.0 209.5 **
mgtA 0.0 0.0 0.0 0.0 3.5 0.0
mgtE 446.5 329.6 385.3 569.9 515.3 612.7 *
yrbG 423.3 530.3 455.7 682.2 443.6 513.8
chaA 48.0 46.8 7.8 82.6 22.7 58.6
lysC 299.9 133.2 274.2 273.6 271.2 214.6
asd 391.3 405.9 293.4 361.0 348.2 312.2
ectA 98.4 64.8 96.7 77.8 29.1 54.7
ectB 200.9 115.1 187.0 203.7 69.3 116.2
ectC 70.6 40.9 74.7 61.2 30.1 36.8
ectD 51.3 10.1 19.8 26.6 6.1 7.1
doeA 190.0 136.3 175.1 165.4 85.1 105.3
doeB 25.4 8.0 9.2 9.2 3.6 8.9
doeC 133.2 59.6 136.6 51.7 22.4 33.5
doeD 184.9 77.9 186.2 75.2 29.7 44.0
codA 0.0 0.0 0.0 0.0 0.0 0.0
betA 162.1 130.3 196.0 133.1 42.6 112.0
betB 33.8 20.7 37.0 68.0 15.2 30.3
otsA 101.2 137.2 123.0 148.3 331.6 188.3
otsB 67.6 125.8 94.6 195.8 305.9 201.6 *
treT 2.0 0.0 2.3 30.6 164.4 108.1
proA 420.4 511.7 328.3 298.7 279.3 250.2
proB 343.0 356.5 281.2 226.2 210.5 207.3 *
proC 312.8 320.2 221.9 212.2 199.6 202.8
TC.BCT 291.5 220.7 284.8 416.6 155.1 307.8
opuA 141.6 112.7 105.1 147.9 143.9 133.1
opuBD 228.1 224.7 151.3 198.2 201.5 182.7
opuC 222.4 172.3 151.8 235.0 184.7 186.2
opuD 66.6 7.2 114.6 118.9 71.3 85.1
proV 270.2 277.1 233.3 383.4 150.1 246.5
proW 236.3 153.8 202.3 325.2 125.1 205.3
proX 287.7 297.8 244.7 551.2 180.7 298.6
bop 0.0 162.8 0.0 6.6 5.9 1.4
hop 0.0 90.8 0.0 5.9 2.9 4.6
sop 7.3 103.4 4.8 7.8 3.6 2.7
uvrA 958.6 1318.7 975.7 1050.3 949.9 1054.9
uvrB 622.5 835.5 586.2 661.8 663.7 649.7
uvrC 682.4 670.5 463.3 492.5 415.3 456.0
uvrD 985.3 821.5 851.1 880.4 777.7 1067.5
mscS 320.6 412.0 376.6 384.9 295.2 370.8
mscL 11.6 3.2 26.5 39.2 62.7 69.1 *
ynaI/mscMJ 96.8 15.5 83.3 320.8 159.1 228.9 *

Figure S4. Heatmap showing the variation in abundance of functional genes involved in carbon cycling pathways across water (W_1, W_4, W_8) and sediment (S_1, S_2, S_3) samples from Lake Barkol. Genes cover carbon fixation (CBB, WL, rTCA), fermentation, methanogenesis, and phototrophy. Marker genes for carbon fixation pathways are indicated with asterisks (*). Statistical differences between groups were calculated using Welch's t-test; $*P < 0.05$, $**P < 0.01$. Gene abundance is shown as counts per million (CPM).

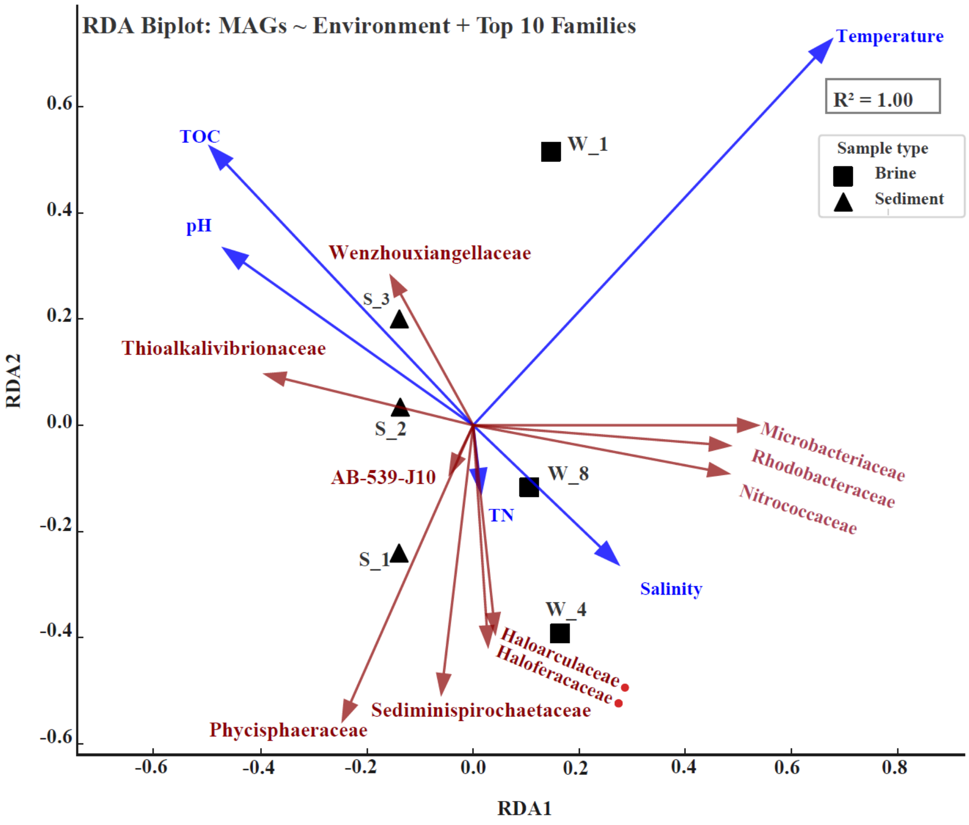

Figure 5S. Redundancy analysis (RDA) biplot showing the relationship between the top 10 most abundant microbial families (brown arrows), environmental variables (blue arrows), and sample types (brine: ■; sediment: ▲) in Lake Barkol. Environmental parameters include salinity, temperature, pH, total organic carbon (TOC), and total nitrogen (TN). The positioning of taxa and samples indicates their correlation with specific environmental gradients. The R^2 value reflects the total variance explained by the model.
